# DDIT3 Directs a Dual Mechanism to Balance Glycolysis and Oxidative Phosphorylation during Glutamine Deprivation

**DOI:** 10.1002/advs.202003732

**Published:** 2021-03-27

**Authors:** Mingyue Li, Rick Francis Thorne, Ronghua Shi, Xu Dong Zhang, Jingmin Li, Jingtong Li, Qingyuan Zhang, Mian Wu, Lianxin Liu

**Affiliations:** ^1^ Heifei National Laboratory for Physical Sciences at the Microscale of USTC CAS Centre for Excellence in Molecular Cell Science the First Affiliated Hospital of University of Science and Technology of China Hefei Anhui 230027 China; ^2^ Translational Research Institute Henan Provincial People's Hospital School of Clinical Medicine Henan University Zhengzhou Henan 450003 China; ^3^ Harbin Medical University Cancer Hospital Harbin Heilongjiang 150081 China

**Keywords:** COQ9, COX4, DDIT3/CHOP, electron transfer chain, glutamine deprivation, glycolysis

## Abstract

Extracellular glutamine represents an important energy source for many cancer cells and its metabolism is intimately involved in maintaining redox homeostasis. The heightened metabolic activity within tumor tissues can result in glutamine deficiency, necessitating metabolic reprogramming responses. Here, dual mechanisms involving the stress‐responsive transcription factor DDIT3 (DNA damage induced transcript 3) that establishes an interrelationship between glycolysis and mitochondrial respiration are revealed. DDIT3 is induced during glutamine deprivation to promote glycolysis and adenosine triphosphate production via suppression of the negative glycolytic regulator TIGAR. In concert, a proportion of the DDIT3 pool translocates to the mitochondria and suppresses oxidative phosphorylation through LONP1‐mediated down‐regulation of COQ9 and COX4. This in turn dampens the sustained levels of reactive oxygen species that follow glutamine withdrawal. Together these mechanisms constitute an adaptive survival mechanism permitting tumor cells to survive metabolic stress induced by glutamine starvation.

## Graphical abstract

Glutamine shortages within the tumor microenvironment necessitate adaptive survival mechanisms in cancer cells. Activation of the transcription factor DNA damage induced transcript 3 under prolonged glutamine deficiency serves to balance cellular adenosine triphosphate energy production and enable cell survival by upregulating glycolysis via transcriptional effects while also dampening oxidative phosphorylation through direct interactions with mitochondrial respiratory chain components.

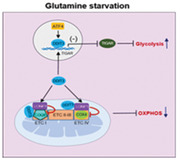

## 1 Introduction

Tumor growth is heavily dependent on glutamine, a nonessential amino acid (NEAA) which is abundant in circulation. Glutamine is a major contributor to the anaplerotic replenishment of the tricarboxylic acid (TCA) cycle, serving as a major source of carbon and nitrogen for the synthesis of proteins, lipids, nucleotides, and amino acids^[^ ^1^, ^2^ ^]^ along with being a precursor of glutamate to facilitate glutathione (GSH) production and redox homeostasis.^[^ ^3^ ^]^ Estimates from tracing analyses show that glutamine contributes at least 50% of the NEAA requirement for protein synthesis by cancer cells.^[^ ^4^ ^]^ Furthermore, anaplerosis for energy production and fatty acid synthesis in cancer cells is highly dependent on glutamine metabolism, especially under conditions of metabolic stress or oncogenic activation.^[^ ^5^, ^6^ ^]^ Notably, glutamine transporters are upregulated among many types of cancer cells,^[^ ^7^, ^8^ ^]^ as is GLS1,^[^ ^9^ ^]^ the mitochondrial enzyme responsible for glutaminolysis (the conversion of glutamine to glutamate). This dependency on glutamine metabolism, often called glutamine addiction, has proposed new targeting opportunities for cancer treatment.^[^ ^10^, ^11^ ^]^

The concentration of glutamine within solid tumors can be heterogenous and frequently insufficient as a result of its heightened utilization and/or restricted blood circulation.^[^ ^12^, ^13^ ^]^ Since glutamine starvation can lead to MYC‐dependent apoptosis as well as enhancing cell death in anticancer drugs treatment,^[^ ^14^, ^15^ ^]^ cancer cells must consequently mount adaptive responses that ensure their survival. Glutamine starvation can induce autophagy,^[^ ^16^ ^]^ as well as inhibition protein synthesis and cell proliferation.^[^ ^17^, ^18^ ^]^ Other coping mechanisms involve enhancing de novo biosynthesis of glutamine or proteolytic scavenging.^[^ ^19^ ^]^ For example, asparagine is able to inhibit endoplasmic reticulum (ER) stress and apoptosis induced by glutamine deprivation, aspartate can inhibit the activity of glutaminase, and cysteine/glutamine exchange creates dependency on glutaminase to maintain glutamine deamination and glutamate production.^[^ ^20^ ^]^ Moreover, in addition to GSH‐mediated oxidative resistance which is essential for eliminating hydrogen peroxide via glutathione peroxidase,^[^ ^21^ ^]^ glutamine also participates in the maintenance of redox homeostasis through nicotinamide adenine dinucleotide phosphate (NADPH) production by malic enzyme.^[^ ^22^ ^]^ Accordingly, glutamine deprivation increases mitochondrial reactive oxygen species (ROS) and decreases GSH levels which can trigger cell death.^[^ ^23^ ^]^ Here, ROS production initially increases 24 h after glutamine deprivation while the levels decrease thereafter,^[^ ^18^ ^]^ with sudden and substantial increases in ROS committing cells to apoptosis.^[^ ^24^, ^25^ ^]^ Instructively, addition of the GSH precursor N‐acetylcysteine (NAC) can reverse glutamine deprivation‐induced cell death.^[^ ^13^ ^]^

The prominent utilization of glucose by tumor cells to generate energy through aerobic glycolysis, as opposed to mitochondrial oxidative phosphorylation (OXPHOS), is the well‐described “Warburg effect” which is responsible for rapid adenosine triphosphate (ATP) generation as well as adaption to the hypoxic tumor environment.^[^ ^26^, ^27^ ^]^ However, the impact of glutamine deprivation stress on glycolysis is controversial, with both reduction^[^ ^28^, ^29^ ^]^ and enhancement of glycolytic flux reported, the latter resulting from increases in the expression of glycolytic enzymes.^[^ ^30^ ^]^ Nonetheless, it is clear that glutamine starvation effects may vary among tumor types, oncogene or tumor suppressor status, epigenetic alterations, and stages of tumor development and tumor environment.^[^ ^31^ ^]^

DDIT3 (DNA damage induced transcript 3) also known as GADD153 (G1 arrest and DNA damage 153) or CHOP (C/EBP homologous protein) is a transcription factor induced by multiple stress conditions including DNA damage, ER stress, hypoxia, and amino acid starvation^[^ ^32^ ^]^ and is best known for regulating genes involved in cell cycle arrest and/or apoptosis.^[^ ^33^ ^]^ Herein we establish that glutamine deprivation increases DDIT3 expression through ATF4‐mediated transcription, with DDIT3 localized to both the cell nucleus and mitochondria. Moreover, we demonstrate that the compartmentalized pools of DDIT3 fulfil discrete mechanistic roles. First, acting in its capacity as a transcription factor, DDIT3 promotes glycolysis through suppressing TIGAR, a negative regulator of glycolysis. Second, mitochondrial localization of DDIT3 attenuates mitochondrial respiration by downregulation of COQ9 and COX4 which impairs electron transport chain (ETC) function. Furthermore, creating a DDIT3 knockout strain of mice established these mechanisms were only induced in wild‐type (WT) but not knockout mice fed a diet lacking glutamine. Thus, in cells deprived of glutamine, vital ATP levels are maintained through glycolysis while mitochondrial respiration is dampened to avoid deleterious production of ROS. The discrete but coordinated actions of DDIT3 therefore contribute to metabolic reprogramming, serving to balance glycolysis against oxidative phosphorylation and allowing cancer cells to survive metabolic stress conditions.

## 2 Results

### 2.1 DDIT3 is Upregulated following Glutamine Deprivation and Promotes Cancer Cell Glycolysis

Previous studies report that short‐term glutamine deprivation increases aerobic glycolysis,^[^ ^30^ ^]^ although not all of the mechanistic details involved are known, particularly the metabolic reprogramming responses of cancer cells to chronic glutamine deprivation. To gain further insights, RNA sequencing analyses (RNA‐seq) were undertaken in hepatocellular carcinoma (HepG2) cells cultured with or without 4 × 10^−3^ m glutamine for 48 h to derive a list of differentially expressed genes (Table S1, Supporting Information). Among the significantly changed genes (log2 fold change > 2 and *p* < 0.01), we selected a short list of eight highly upregulated genes and used quantitative polymerase chain reaction (qPCR) to verify their expression changes following glutamine deprivation. Indeed, significant increases in the levels of *DDIT3*, *CHAC1*, *STC2*, *TRIB3*, *ASNS*, *PCK2*, *GDF15*, and *OSGIN* were recorded in HepG2 cells as well as HCT116 colon and MCF7 breast carcinoma cells (Figure S1A, Supporting Information).

As a preliminary means to explore the contribution of these genes to glycolysis, we individually knocked down each in HepG2 cells. In particular, depletion of DDIT3 strikingly decreased culture medium acidification (Figure S1B, Supporting Information), indicative of altered glycolysis resulting from decreased lactate secretion.^[^ ^34^ ^]^ Repeating the assay using three independent shRNAs targeting DDIT3 in three different cell types (HepG2, HCT116, and MCF‐7) revealed similar effects, indicating that the delayed acidification resulted from on‐target effects against DDIT3 and moreover, that the putative effects on glycolysis were not cell line dependent (Figure S1C, Supporting Information). Toward further dissecting the observed DDIT3 phenotype, we utilized CRISP‐CAS9 technology to derive HepG2 cells completely lacking DDIT3 expression, herein referred to as DDIT3‐KO. We then sought to verify the effects of DDIT3 on lactate production resulted from effects on glycolysis.

**Figure 1:**
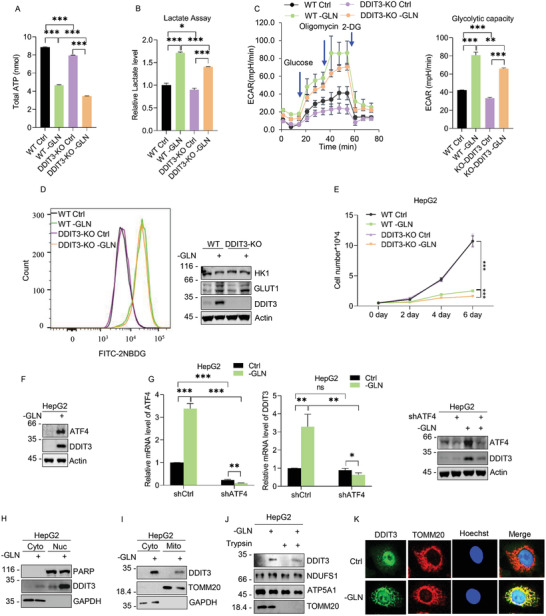
DDIT3 is upregulated under glutamine deprivation and promotes cancer cell glycolysis. A) Intracellular ATP levels determined in WT and CRISPR‐CAS9 modified HepG2 cells lacking DDIT3 (KO) cultured with or without 4 × 10^−3^ m glutamine for 48 h (left). B) Extracellular lactate production determined in WT and CRISPR‐CAS9 modified HepG2 cells lacking DDIT3 (KO) cultured with or without 4 × 10^−3^ m glutamine for 48 h. C) WT and DDIT3‐KO HepG2 cells were subjected to glycolytic stress analyses using the Seahorse XF analyzer in response to glucose, oligomycin, and 2‐DG in the presence (Ctrl) and absence of glutamine (−GLN). Responses are expressed as the extracellular acidification rate (ECAR) (left). AUC (area under curve) measurements of ECAR from (left) were used to derive measured glycolytic capacity (right). D) Glucose uptake of WT and DDIT3‐KO HepG2 cells cultured with or without 4 × 10^−3^ m glutamine for 48 h measured with 2‐NBDG (left). Expression of HK1, GLUT1, and DDIT3 determined by Western blot (right). E) Growth of HepG2 cells as per (A) measured as total cell numbers determined over 6 days. F) Expression of ATF4 and DDIT3 in HepG2 cells determined by Western blot after culture in medium with (+) or without (−) 4 × 10^−3^ m glutamine (GLN) for 48 h. G) HepG2 cells transduced with control (pLKO.1) or shRNAs targeting ATF4 (shATF4#5; Table S1, Supporting Information) were cultured in the presence (Ctrl) or absence of glutamine (GLN) for 48 h. Relative mRNA (left) and protein (right) levels of ATF4 and DDIT3 were determined by qPCR and Western blot, respectively. H,I) Comparison of the subcellular distribution of DDIT3 in H) nuclear and cytosolic fractions or I) mitochondrial and cytosolic fractions of HepG2 cells determined by Western blotting after 48 h culture under glutamine replete or deprivation conditions. Blotting against PARP, GAPDH, and TOMM20 served as nuclear, cytoplasmic, and mitochondrial markers, respectively. J) Mitochondrial fractions from (I) were analyzed by Western blot without (−) or with (+) trypsin treatment to proteolyze the mitochondrial outer membrane. The expression of DDIT3 is compared with proteins known to localize to the inner (NDUFS1, ATP5A1) and outer (TOMM20) mitochondrial membranes. K) Confocal imaging of DDIT3 and TOMM20 expression in HepG2 cells under control or glutamine deprivation conditions. A–K) Data represent three independent experiments. A–C,E,G) Data are mean ± SD, *n* = 3, *p < 0.05; **p < 0.01; ***p < 0.001; ns, not significant, two‐tailed paired Student's *t* test.

Comparative cellular ATP and secreted lactate measurements were first conducted in HepG2 parental versus DDIT3‐KO cells. Subjecting parental HepG2 cells to glutamine deprivation resulted in decreased levels of ATP and increased levels of secreted lactate (**Figure** **1**A,B), consistent with a metabolic shift from OXPHOS toward aerobic glycolysis. In comparison, ATP levels in DDIT3‐KO cells were reduced compared to WT cells, modestly under basal conditions but more substantially following glutamine deprivation with accompanying dampening of changes in lactate levels. Corroborating this notion, Seahorse XF assays measuring the extracellular acidification rate (ECAR), a proxy measure of glycolytic flux, showed that glutamine deprivation stimulated glycolysis in HepG2 cells whereas both basal and glutamine deprivation responses were dampened in DDIT3‐KO cells (Figure 1C). Moreover, analysis using 2‐NBDG uptake assay showed glutamine deprivation stimulated glucose uptake capacity which was associated with the increased levels of the glucose transporter GLUT1 (Figure 1D). However, no changes were observed in hexokinase 1 (HK1), one of the rate‐limiting enzymes in glycolysis. Examination of long‐term growth showed there were no significant differences between parental and DDIT3‐KO HepG2 cells under glutamine replete conditions, indicating the reduction in ATP levels did not impact growth, presumably because of the abundant exogenous energy source. However, glutamine deprivation caused profound growth inhibition in parental cells which was further inhibited by DDIT3‐KO (Figure 1E). Together these results indicate that DDIT3 functions to promote glycolysis, providing tangible benefits for the growth of cells compromised by glutamine starvation.

Among a range of stressors, glutamine deprivation is known to upregulate DDIT3,^[^ ^35^ ^]^ but the underlying mechanism driving its expression under these conditions is not clear. The transcription factor ATF4 transactivates DDIT3 during ER stress^[^ ^36^ ^]^ as well as amino acid deprivation^[^ ^37^ ^]^ and we assessed if it was also responsible for driving DDIT3 transcription during glutamine deprivation. Indeed, ATF4 along with DDIT3 were robustly upregulated in response to glutamine deprivation (Figure 1F) and moreover, knockdown of ATF4 prevented the induction of DDIT3 mRNA and protein in cells deprived of glutamine (Figure 1G). Notably, ATF4 is itself known to be regulated by GCN2, a sensor of amino acid levels that is activated by amino acid deprivation.^[^ ^38^, ^39^ ^]^ The levels of total GCN2 protein in both WT and DDIT3‐KO HepG2 cells were marginally reduced by glutamine deprivation, while in contrast, activated GCN2 (phospho‐Thr899‐GCN2) levels were markedly elevated (Figure S2A, Supporting Information). Instructively, depleting GCN2 using shRNA completely ablated the increased levels of both ATF4 and DDIT3 induced by glutamine deprivation (Figure S2B, Supporting Information). Together with the preceding data, this indicates a canonical sequence where glutamine deprivation activates GCN2 to upregulate ATF4 which in turn triggers transcriptional increases in DDIT3.

Next, we turned to consider the localization of DDIT3 in cells. Studies of ER stress responses reported the prominent accumulation of a cytoplasmic pool of DDIT3,^[^ ^32^ ^]^ although its function is not fully known. Analysis of the subcellular localization of DDIT3 in HepG2 cells revealed a large proportion of DDIT3 was associated with nuclear fractions but with some DDIT3 evident in cytoplasmic fractions in response to glutamine deprivation (Figure 1H). Further fractionation experiments delineated that the increased DDIT3 levels following glutamine deprivation belonged to both cytosolic and mitochondria pools (Figure 1I). Confirming the specificity of this result, treating isolated mitochondrial fractions with trypsin failed to proteolyze the DDIT3‐reactive band (Figure 1J), indicating that a fraction of DDIT3 translocated into the inner mitochondria membrane or intermembrane space. Consistently, analysis by confocal microscopy revealed that glutamine deprivation promoted substantial increases in DDIT3 cytoplasmic staining with a strong overlap with the mitochondrial marker, TOMM20 (Figure 1K).

As a corollary to these observations, we considered if manipulating DDIT3 also impacted lipid uptake and lipolysis, particularly in the context of glutamine deprivation where it has been reported that HepG2 cells accumulate lipid and exhibit protein expression changes associated with lipid metabolism.^[^ ^40^ ^]^ Measuring intracellular neutral lipids using Nile Red showed glutamine starvation induced higher lipid accumulation in WT cells with DDIT3‐KO cells showing comparatively higher lipid accumulation under basal conditions (Figure S2C, Supporting Information). In parallel, we measured the expression of the lipid transporters FATP2 and CD36 together with ATGL and ACSL1a, which play roles in lipid catabolism. Glutamine starvation induced FATP2 protein increases in WT cells, while FATP2 levels were relatively higher in DDIT3‐KO cells and were not affected by changing glutamine conditions. ACSL1a were similarly regulated to FATP2 but in contrast, there were no apparent changes in CD36 or ATGL (Figure S2D, Supporting Information). Thus, the increased lipid accumulation tracks with the increased expression of FATP2 and ASCL1a.

Taken together, these results indicate that DDIT3 is remarkably upregulated through ATF4 transcription, and in addition to the anticipated nuclear localization, a notable pool of DDIT3 can translocate to the inner membrane of mitochondria. Moreover, while DDIT3 contributes to the basal maintenance of glycolytic flux in HepG2 cells, this role did not appear critical to long‐term cell growth under normal conditions. Rather, a more critical impact occurs during glutamine deprivation where a large proportion of the glycolytic actuation requires DDIT3, and in DDIT3's absence, glycolysis is notably abridged. On this basis, we sought to define DDIT3 functions, particularly the contributions of the nuclear and mitochondrial pools.

### 2.2 DDIT3 Promotes Glycolysis via Transcriptional Suppression of TIGAR

**Figure 2:**
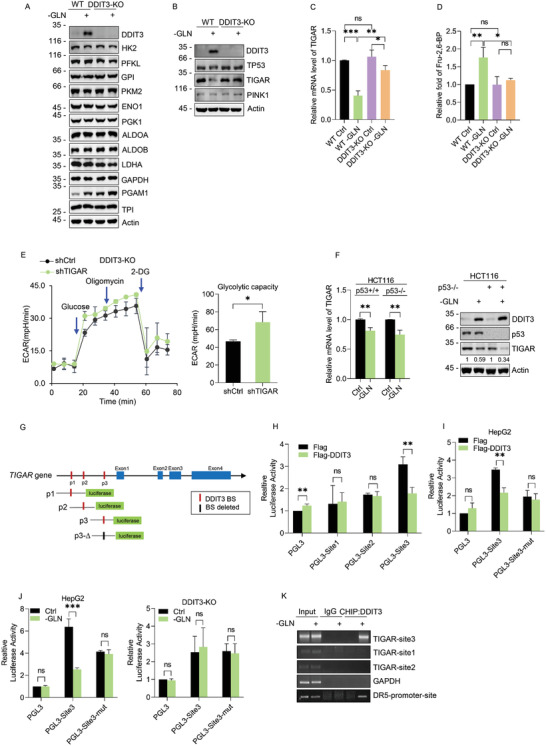
DDIT3 promotes glycolysis via transcriptional suppression of TIGAR. A) The expression of key glycolytic pathway enzymes measured in WT versus DDIT3‐KO HepG2 cells cultured with (+) or without (−) 4 × 10^−3^ m glutamine for 48 h by Western blot. Actin served as a loading control. B) Expression of DDIT3, p53, TIGAR, and PINK1 determined by Western blot and C) relative mRNA levels of TIGAR determined by qPCR in WT and DDIT3‐KO HepG2 cells treated as per (A). D) Relative cellular levels of Fru‐2‐6‐BP determined by LC‐MS in WT and DDIT3‐KO HepG2 cells treated as per (A). E) Seahorse XF assay comparing ECAR in DDIT3‐KO HepG2 cells transfected with control (PLKO.1) or shRNAs targeting TIGAR (shTIGAR) (left). AUC measurements of ECAR were used to derive measured glycolytic capacity (right). F) Relative mRNA level of TIGAR was determined by qPCR (left) and Western blot analyses comparing the expression of DDIT3 and TIGAR in p53 WT versus p53 KO HCT116 cells after culture with (+) or without (−) 4 × 10^−3^ m glutamine (GLN) for 48 h (right). G) Predicted DDIT3 binding sites present in the proximal promoter region of *TIGAR* were utilized in the design of pGL3‐based luciferase reporter constructs (Sites p1, p2, p3). H) DDI3T‐KO HepG2 cells were transfected for 24 h with pGL3 (control) or the individual *TIGAR* promoter reporters in combination with Renilla and/or empty expression vector (Flag) or Flag‐DDIT3. Normalized the luciferase activity of three promoter sites (pGL3‐Site1, ‐Site2, ‐Site3) determined from dual luciferase assays. I) Luciferase reporter assays were conducted as per (H) after transfecting WT HepG2 cells with pGL3, pGL3‐Site3, or pGL3‐Site3 mutant vectors after transfection with Flag control or Flag‐DDIT3 vectors. J) Luciferase reporter assays were conducted after transfecting WT HepG2 cells (left) or DDIT3‐KO HepG2 cells (right) with pGL3, pGL3‐Site3, or pGL3‐Site3 mutant vectors in the presence (Ctrl) or absence of glutamine (GLN). K) Chromatin immunoprecipitation (ChIP) assays performed in DDIT3‐KD HepG2 cells against negative control IgG, anti‐DDIT3, or DR5 antibodies (positive control). Semiquantitative PCR was performed using ChIP primers indicated as the red bar in (G). A–F,H–K) Data represent three independent experiments. C,D,F (left),H–J) Data are mean ± SD, *n* = 3, *p < 0.05; **p < 0.01; ***p < 0.001; ns, not significant, two‐tailed paired Student's *t* test.

We first investigated if expression changes in glycolytic pathway enzymes could explain alterations in glycolytic flux either resulting from glutamine deprivation or DDIT3 knockdown. However, analyses of the 11 glycolytic pathway enzymes as well as lactate dehydrogenase A (LDHA) showed that only phosphoglycerate mutase 1 (PGAM1) was increased in HepG2 cells as a result of glutamine deprivation or DDIT3‐KO (**Figure** **2**A). Since the expression of an individual enzyme cannot affect the efficiency of the whole cascade,^[^ ^41^ ^]^ this proposes DDIT3 modulates glycolysis independently of effects on the primary levels of the enzymes involved.

Glycolytic regulation is also known to occur via other means with two established negative regulators being TIGAR (p53‐induced glycolysis and apoptosis regulator) and PINK1 (PTEN‐induced kinase 1). TIGAR can lower intracellular levels of fructose‐2,6‐bisphosphate (Fru‐2,6‐BP), resulting in pentose phosphate pathway activation and NADPH production,^[^ ^42^ ^]^ whereas *PINK1* negatively regulates glycolysis via blocking HIF1*α* stabilization.^[^ ^43^ ^]^ Examining the protein levels of both TIGAR and PINK1 in HepG2 cells revealed that TIGAR expression was decreased during glutamine deprivation while the levels of PINK1 displayed no obvious changes (Figure 2B). Instructively, TIGAR levels were not decreased in DDIT3‐KO cells, suggesting that DDIT3 is involved in the downregulation of TIGAR in glutamine‐deprived cells. Consistently, qPCR measurements showed TIGAR mRNA levels were reduced during glutamine deprivation whereas knockout of DDIT3 served to reverse this reduction (Figure 2C). Since TIGAR bisphosphatase activity converts Fru‐2,6‐BP to Fru‐6‐P, it was anticipated that reduced TIGAR expression would lead to Fru‐2,6‐BP accumulation. Indeed, quantitative liquid chromatography–mass spectrometry (LC‐MS)‐based metabolomic analysis revealed that Fru‐2,6‐BP levels were increased in response to glutamine deprivation in HepG2 but not in DDIT3‐KO cells (Figure 2D). Moreover, knockdown of TIGAR in DDIT‐KO cells increased glycolytic flux (Figure 2E), proposing that DDIT3‐regulated glycolysis is required by TIGAR.

TIGAR is a well‐known transcriptional target of p53^[^ ^42^ ^]^ and the availability of p53 knockout and replete HCT116 cells allowed us to evaluate the contribution of p53 to TIGAR expression during glutamine deprivation. The levels of TIGAR mRNA decreased in response to glutamine withdrawal to a similar extent in both p53 WT and KO cells (Figure 2F, left) and the levels of TIGAR protein diminished accordingly (Figure 2F, right). This indicates the reduced TIGAR expression during glutamine deprivation primarily results from transcriptional downregulation independent of p53.

Given the impact of DDIT3 on TIGAR expression (Figure 2B), we next considered how it was involved in the transcriptional downregulation of TIGAR during glutamine deprivation. Analysis of the *TIGAR* genomic region for potential DDIT3 binding motifs (JASPAR database) revealed three high scoring binding sites (BSs) embedded in the *TIGAR* proximal promoter (Figure 2G, top). Luciferase reporter constructs designed to individually test the activity of each BS (Figure 2G, bottom) showed that only BS3 was responsive to expression of ectopic Flag‐DDIT3 (Figure 2H), and decisively, this activity was abrogated when the binding sequence was deleted (Figure 2I). Instructively, the reporter activity of the BS3 construct was inhibited following glutamine deprivation (Figure 2J, left), but no changes occurred in response to glutamine deprivation when these assays were conducted in DDIT3 knockdown cells (Figure 2J, right). Moreover, chromatin immunoprecipitation (ChIP) assays showed that BS3 fragment was selectively recovered in DDIT3 immunoprecipitations (Figure 2K). Thus, DDIT3 directly inhibits the transcription of TIGAR during glutamine deprivation via occupying the *TIGAR* promoter.

### 2.3 DDIT3 Suppresses Mitochondrial Respiration during Glutamine Deprivation

Glutamine represents a major energy source for transformed cells via its contribution to mitochondrial oxidative phosphorylation,^[^ ^44^ ^]^ and consistently our experiments demonstrated glutamine starvation led to profound increase in cellular ATP levels. Under these conditions, DDIT3 contributes to cellular energy production through promoting glycolytic flux but at the same time, the translocation of DDIT3 to mitochondria suggested an additional role. On this basis, we examined the effects of DDIT3 on oxidative phosphorylation.

**Figure 3:**
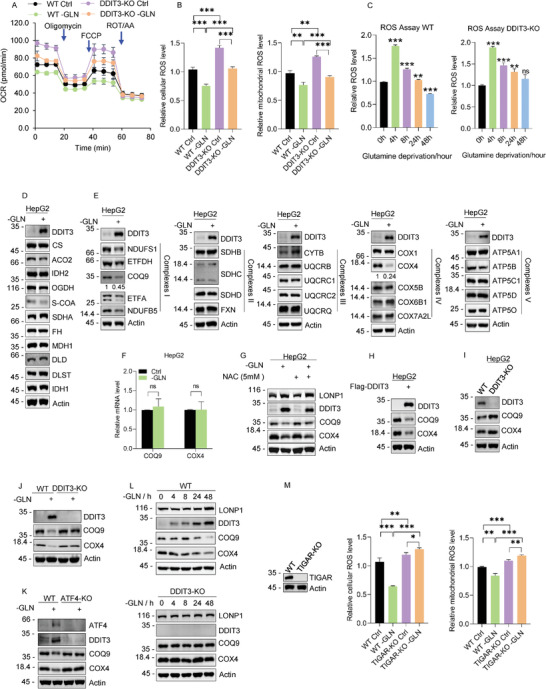
DDIT3 suppresses mitochondrial respiration during glutamine deprivation. A) Seahorse XF assay measuring the OCR of WT and DDIT3‐KO HepG2 cultured with 4 × 10^−3^ m (Ctrl) or without (−GLN) glutamine for 48 h. B) The levels of cellular ROS (left) and mitochondrial ROS (right) are compared in cells from (A). C) Cellular ROS levels determined in DDIT3‐WT (left) and DDIT3‐KO HepG2 cells (right) cultured under glutamine deprivation for 0, 4, 8, 24, 48 h. D) Western blot analysis comparing the majority of TCA cycle enzymes expression levels with 4 × 10^−3^ m (+) or without (−) glutamine treatment for 48 h. E) Western blotting against core components of oxidative phosphorylation in WT HepG2 cells cultured with 4 × 10^−3^ m (+) or without (−) glutamine for 48 h. Analyses are divided into electron transport chain (ETC) complexes I–V including DDIT3. Actin served as a loading control throughout. Image J software was used to quantify the band density. F) qRT‐PCR analyses of COQ9 and COX4 in HepG2 cultured with 4 × 10^−3^ m (+) or without (−) glutamine for 48 h. G) HepG2 cells were cultured with or without 4 × 10^−3^ m glutamine for 24 h before the addition of 5 × 10^−3^ m NAC for a further 24 h as indicated. Western blot analyses then compared the relative levels of DDIT3, COQ9, and COX4. H) Western blot analyses comparing the relative levels of DDIT3, COQ9, and COX4 in HepG2 cells 24 h after transfection with Flag control or Flag‐DDIT3 vectors H), I) in WT versus DDI3T KO cells grown under normal conditions or J) subjected to glutamine deprivation (−GLN) for 48 h. K) Western blot analyses of ATF4, DDIT3, COQ9, and COX4 in WT or ATF4‐KO HepG2 cells grown under normal conditions or glutamine deprivation (−GLN) for 48 h. L) LONP1, DDIT3, COQ9, and COX4 protein levels were compared under glutamine deficiency for 0–48 h in WT‐DDIT3 (top) versus DDIT3‐KO HepG2 cells (bottom). M) The levels of cellular ROS (left) and mitochondrial ROS (right) in WT and TIGAR‐KO HepG2 cells cultured with or without 4 × 10^−3^ m glutamine for 48 h. TIGAR protein knockout efficiency shown far right. A–M) Data represent three independent experiments. A–C,F,M) Data are mean ± SD, *n* = 3, *p < 0.05; **p < 0.01; ***p < 0.001; ns, not significant, two‐tailed paired Student's *t* test.

Mitochondrial stress tests were applied to measure the oxygen consumption rate (OCR), a proxy measure indicative predominantly for mitochondrial respiration. Analysis of WT HepG2 cells showed, as expected, that glutamine deprivation leads to decreased OCR measurements. Strikingly, repeating these assays in DDIT3‐KO cells revealed that comparably higher OCR measurements compared to WT cells (**Figure** **3**A). In support, measurements of ROS phenocopied the OCR changes with total and mitochondrial ROS levels being inhibited following glutamine deprivation but nonetheless ROS levels were significantly higher in DDIT3‐KO cells (Figure 3B). Consistent with previous reports showing the effects of glutamine starvation,^[^ ^18^ ^]^ cellular ROS levels changed in a time‐dependent manner, peaking at 4 h and diminishing thereafter to be lower than control levels at 48 h (Figure 3C, left). However, while glutamine starvation also stimulates ROS in DDIT3‐KO cells, higher levels than parental HepG2 cells are sustained during the temporary response (Figure 3C, right). Together these data indicate that during glutamine starvation, oxidative phosphorylation was inhibited by DDIT3 and that in the absence of DDIT3 leads to sustained levels of cellular ROS.

Examination of each of the enzymes involved in the TCA cycle revealed no significant variations in cells subjected to glutamine deprivation (Figure 3D). Rather we considered if there were changes in ETC that could account for the downregulation of mitochondrial respiratory activity following glutamine deprivation. Indeed, there were overall reductions the levels of key elements of ETC complexes I, II, III, IV, and V, especially the complex I component, COQ9, and the complex IV component, COX4 (Figure 3E). Notably the reduction in COQ9 and COX4 proteins were not dependent changes in their respective mRNA levels (Figure 3F), ruling out the likely involvement of transcriptional changes. We also considered whether changes in mitochondrial ROS levels resulting from glutamine starvation caused changes in COQ9 and COX4 levels. However, treating WT HepG2 cells with the ROS scavenger NAC did not affect the glutamine‐starvation induced increases in LONP1 and DDIT3 and nor did it block the resulting decreases in COQ9 and COX4 (Figure 3G). Since the reductions of COX4 and COQ9 during glutamine starvation were not influenced by ROS production, we therefore turned to consider the direct involvement of DDIT3.

Strikingly, ectopic expression of Flag‐DDIT3 suppressed the levels of COQ9 and COX4 while conversely, COQ9 and COX4 levels were elevated in DDIT3‐KO cells (Figure 3H,I, respectively). Instructively, examination of glutamine deprivation responses in ATF4‐KO HepG2 cells showed, as anticipated, KO of ATF4 prevented increases in DDIT3 induced by glutamine starvation as well as the reductions in the levels of COQ9 and COX4 (Figure 3K). Consistently, the downregulation of COQ9 and COX4 evident in HepG2 cells following glutamine deprivation was absent after DDIT3 knockout (Figure 3L). Moreover, COQ9 and COX4 proteins displayed time‐dependent decreases in expression in response to glutamine starvation, but this response was completed nullified in DDIT3‐KO cells (Figure 3L). Finally, given TIGAR has antioxidant properties,^[^ ^45^, ^46^ ^]^ and was negatively regulated by DDIT3 under glutamine deprivation (Figure 2B,C), we evaluated the contribution of TIGAR to ROS changes in this setting. Knockout of TIGAR increased cellular and mitochondrial ROS levels over WT cells under normal culture conditions, while glutamine starvation further elevated ROS levels (Figure 3M). That TIGAR KO moderately drives up ROS is concordant with the limited effects of DDIT3 on TIGAR under basal conditions (Figure 2B,C). Furthermore, cells lacking TIGAR likely exhibit higher ROS under glutamine deprivation stress (Figure 3M). Collectively these results propose that DDIT3 attenuates mitochondrial respiration following glutamine deprivation by downregulation of ETC complex I and IV components, particularly COQ9 and COX4, through an unidentified post‐translational regulatory mechanism. We next focused on discerning the mechanistic basis of DDIT3's role in the mitochondria.

### 2.4 DDIT3 Regulates COQ9 and COX4 through LONP1

**Figure 4:**
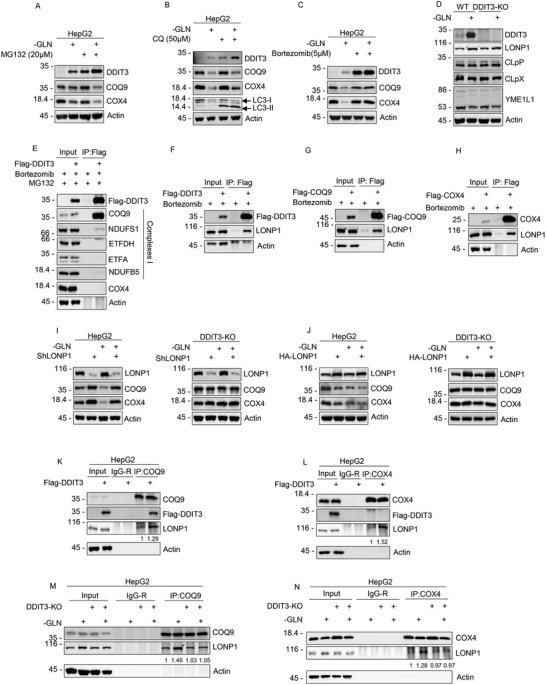
DDIT3 regulates COQ9 and COX4 through LONP1. A) Western blotting of DDIT3, COQ9, and COX4 in HepG2 cells cultured 48 h with or without 4 × 10^−3^ m glutamine and then treated with or without 20 × 10^−6^ m MG132 for 4 h. Actin was used throughout as a loading control. B) Western blotting of DDIT3, COQ9, COX4, and LC3 after culture with or without 4 × 10^−3^ m glutamine for 48 h and then treatment with or without 50 × 10^−6^ m CQ for 6 h. C) Western blotting of DDIT3, COQ9, and COX4 after culture with or without 4 × 10^−3^ m glutamine for 48 h and then treatment with or without 5 × 10^−6^ m Bortezomib for 8 h. D) Western blotting of DDIT3, LONP1, CLpP, CLpX, and YMEL1 in either WT or DDIT3‐KO HepG2 cells cultured with 4 × 10^−3^ m (+) or without (−) glutamine for 48 h. E) Co‐immunoprecipitation analyses conducted on Flag control or Flag‐DDIT3 transfected 293T cells pretreated with 20 × 10^−6^ m MG132 (−4 h) and 5 × 10^−6^ m Bortezomib (−8 h). Anti‐Flag (M2) precipitated samples were subjected to Western blotting against ETC complex I components (COQ9, NDUFS1, ETFDH, ETF, NDUFB5) along with the complex IV component, COX4. Blotting against Flag was used to reveal transfected DDIT3. F–H) 293T cells transfected with either F) Flag control or Flag‐DDIT3, or G) Flag‐COQ9 or H) Flag‐COX4 individually were treated with 5 × 10^−6^ m Bortezomib for 8 h and immunoprecipitation performed using anti‐Flag (M2) beads. Precipitates were immunoblotted with anti‐Flag, and the co‐precipitated LONP1 is indicated. I) Western blot analysis of LONP1, COQ9, and COX4 in WT (left) or DDIT3‐KO HepG2 cells (right) transduced with control (pLKO.1) or LONP1 targeting shRNA and cultured with 4 × 10^−3^ m (+) or without (−) glutamine for 48 h. J) Western blot analyses of LONP1, COQ9, and COX4 in WT (left) or DDIT3‐KO HepG2 cells (right) transfected for 24 h with control HA or HA‐LONP1 and cultured with 4 × 10^−3^ m (+) or without (−) glutamine for 48 h. K) HepG2 cells transfected with either Flag control or Flag‐DDIT3 were added protease inhibitor cocktail 1:25 before cell lysis and immunoprecipitations performed using control IgG (IgG‐R) or anti‐COQ9. Precipitates were immunoblotted for COQ9, anti‐Flag, and LONP1. L) HepG2 cells transfected with either Flag control or Flag‐DDIT3 were added protease inhibitor cocktail 1:25 before cell lysis and immunoprecipitations performed using control IgG (IgG‐R) or anti‐COX4. Precipitates were immunoblotted for COX4, anti‐Flag, and LONP1. M) Immunoprecipitations performed using control IgG (IgG‐R) or anti‐COQ9 in WT or DDIT3‐KO HepG2 cells cultured with or without 4 × 10^−3^ m glutamine for 48 h. Precipitates were immunoblotted for COQ9 and LONP1. Protease inhibitor cocktail was added before cell lysis. N) Immunoprecipitations performed using control IgG (IgG‐R) or anti‐COX4 in WT or DDIT3‐KO HepG2 cells cultured with or without 4 × 10^−3^ m glutamine for 48 h. Precipitates were immunoblotted for COX4 and LONP1. Protease inhibitor cocktail was added before cell lysis. A–N) Data represent three independent experiments. K–N) Image J software was used to quantify the band density.

To evaluate likely post‐translational mechanisms involved in the DDIT3‐mediated regulation of COQ9 and COX4, we employed inhibitor of proteasomal activity MG132 and Bortezomib, as inhibitor of hydrolases together with chloroquine that inhibits mitophagy. HepG2 cells cultured under control or glutamine deprivation conditions were pretreated with either MG132 (4 h),^[^ ^47^ ^]^ Bortezomib (8 h; which also inhibits mitochondrial matrix proteases),^[^ ^48^ ^]^ or chloroquine (6 h)^[^ ^49^ ^]^ according to their optimal pharmacological characteristics. Comparative measurements of protein levels showed Bortezomib but not MG132 or chloroquine rescued the reduction in COQ9 and COX4 levels resulting from glutamine deprivation (**Figure** **4**A–C). Since Bortezomib is known to inhibit the AAA+ superfamily proteases (LONP1, YME1L1, CLpP, and CLpX), the main protease enzymes in mitochondria,^[^ ^50^ ^]^ we investigated their expression in both parental HepG2 cells and DDIT3‐KO cells before and after glutamine deprivation. However, Western blot analyses indicated there were no expression changes evident in either YME1L1, CLpP, and CLpX observed although a modest increase occurred in LONP1 levels during glutamine deprivation in both the presence and absence of DDIT3 (Figure 4D). Together these data suggest that AAA+ superfamily proteases may be involved in the downregulation of COQ9 and COX4 but DDIT3 is not responsible for any wholesale changes in expression of these enzymes. Given the specific actions of DDIT3 against COQ9 and COX4 (Figure 3E), we hypothesized that DDIT3 may act as chaperone to direct these for proteolytic destruction.

On this basis, we evaluated if there were any direct interactions between DDIT3 and either COQ9 or COX4. Taking advantage of the stabilization actions of Bortezomib, co‐immunoprecipitation (co‐IP) analyses were conducted between ectopically expressed Flag‐DDIT3 and endogenous Complex I components as well as COX4. Notably, there was a robust recovery of COQ9 and to a lesser extent NDUFS1 in Flag‐DDIT3 precipitates but conversely there were no interactions evident with COX4 (Figure 4E). To find the likely respective interactions between DDIT3, COQ9, COX4, and LONP1, we conducted co‐IP experiments, this time using ectopically expressed Flag‐tagged DDIT3 along with Flag‐COQ9 and Flag‐COX4. As expected, LONP1 was recovered within Flag‐DDIT3, Flag‐COQ9, and Flag‐COX4 precipitates (Figure 4F–H). These findings propose that COQ9 and COX4 may serve as hydrolytic substrates of LONP1, although the ability of DDIT3 to bind COQ9 but not COX4 suggests some mechanistic differences.

To validate the role of LONP1 and its intersection with DDIT3 function, we treated parental HepG2 and DDIT3‐KO cells with shRNA against LONP1 (shLONP1) and measured the levels of COQ9 and COX4 under both normal and glutamine‐deprived culture conditions. Irrespective of culture conditions, the levels of both COQ9 and COX4 increased following depletion of LONP1 in parental cells while in contrast, shLONP1 treatment in DDIT3‐KO cells had no effect on either COQ9 or COX4 levels (Figure 4I). This suggests that LONP1 is responsible for the degradation of both COQ9 and COX4 but that DDIT3 expression is required for this to occur. To verify this, we alternatively overexpressed LONP1 and measured the impact on COQ9 or COX4 levels. Ectopic HA‐LONP1 expression did not strongly impact the levels either COQ9 or COX4 in parental HepG2 cells under basal conditions but it did accentuate their reduced expression during glutamine deprivation (Figure 4J). Nevertheless, in the absence of DDIT3, HA‐LONP1 had no effect on COQ9 or COX4 levels, again showing that DDIT3 is essential for the actions of LONP1.

Finally, having identified LONP1 as the protease responsible for destruction of COQ9 and COX4 under glutamine deprivation conditions, we returned to refine the hypothesis that DDIT3 acts as chaperone for COQ9 and COX4. We first assessed the potential of DDIT3 to augment associations between either COQ9 or COX4 and LONP1 in parental HepG2 cells under normal culture conditions. Indeed, co‐IP analyses revealed that the levels of LONP1 co‐precipitated with either endogenous COQ9 or COX4 were increased when cells were transfected with Flag‐DDIT3 (Figure 4K,L, respectively). As anticipated from prior experiments, Flag‐DDIT co‐precipitated with COQ9 but not COX4. We then observed the impact of both glutamine deprivation and DDIT‐KO on the levels of co‐precipitated LONP1‐COQ9 and LONP1‐COX4. In parental cells, there were increases in the amount of LONP1 co‐precipitated with both COQ9 and COX4 in response to glutamine deprivation whereas there were no increases in either LONP1‐COQ9 or LONP1‐COX4 complexes in DDIT3‐KO cells (Figure 4M,N, respectively). Collectively, these indicate that DDIT3 promotes LONP1‐hydrolysis of COQ9 and COX4 via facilitating their interactions with LONP1.

### 2.5 Physiological Conservation of DDIT3 in Mice

**Figure 5:**
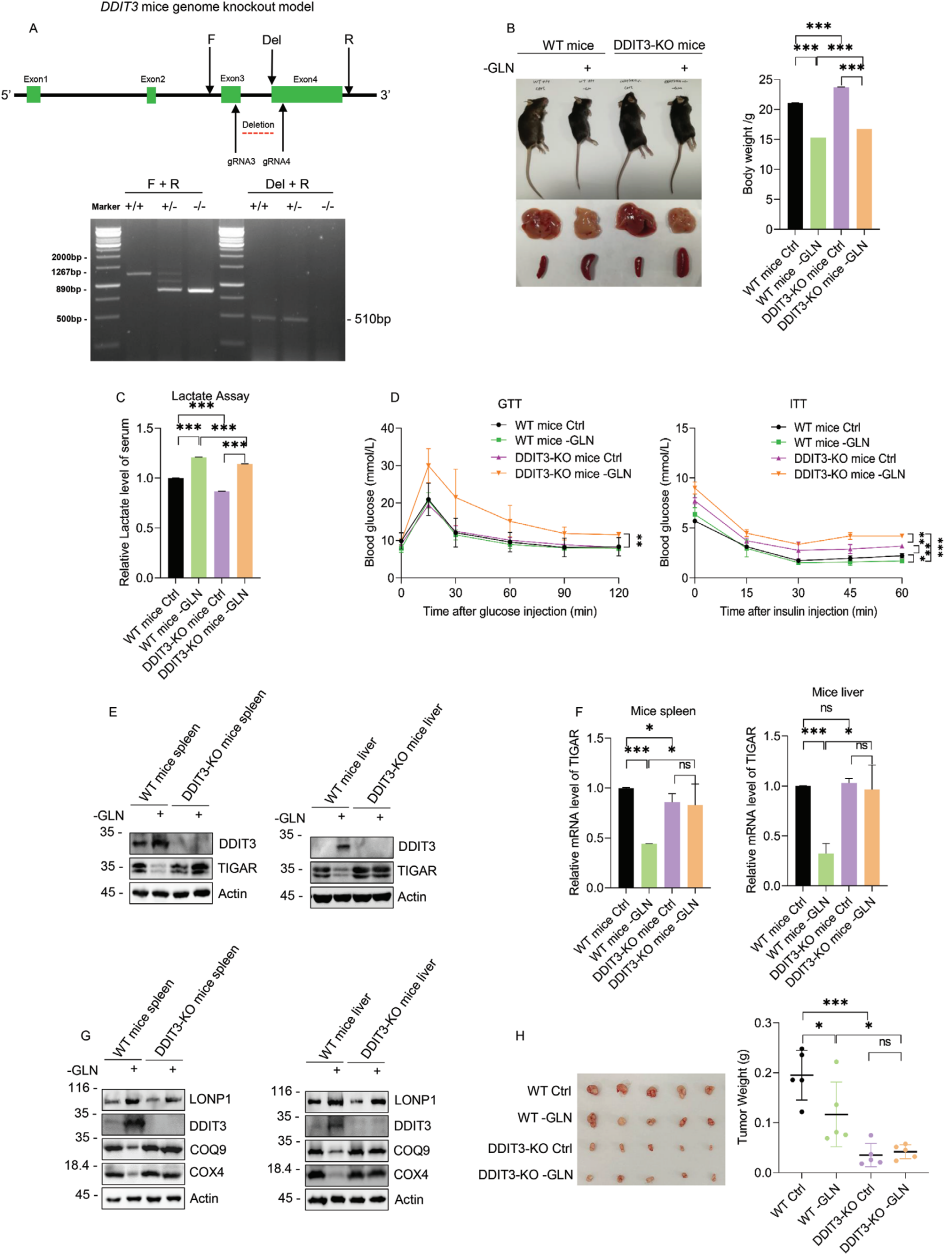
Physiological conservation of DDIT3 in mice. A) Illustration of the CRISPR‐CAS9‐based *DDIT3* gene‐knockout strategy in mice showing the location of the CRISPR guide sequences along with the PCR primers used for the genotyping assays (upper). Dual PCR reactions employing Forward (F)–Reverse (R) primers and the Deletion (Del)–Reverse primers enable validation of wildtype (+/+), heterozygous (+/−), and DDIT‐KO (−/−) mice (lower). B) Representative appearance of WT and DDIT3‐KO body, liver and spleen (left) and body weight comparisons (right) in 12 week old mice maintained on a normal or glutamine‐deficient (−GLN) diets. C) Serum lactate measurements from mice in (B) after 9 weeks post‐weaning maintenance on a normal or glycine‐deficient (−GLN) diet. D) GTT and ITT assays in 12 weeks old WT or DDIT3‐KO mice maintained on normal (+GLN) or glutamine‐deficient (−GLN) diets. E,F) The levels of E) TIGAR protein and F) mRNA were determined in the spleen (left) and liver (right) tissues from mice as per (B) using Western blotting and qPCR, respectively. G) Western blot analyses of LONP1, DDIT3, COQ9, and COX4 in spleen (left) or liver tissues (right) as per (E). H) WT or DDIT3‐KO HepG2 cells were grown as xenografts in nu/nu mice fed continuously with a normal or glutamine‐deficient diet for 4 weeks. Tumors were excised (left) and weighed (right). B–D,F,H) *n* = 8, 3, 3, 3, and 5 mice per group, respectively. Data are mean ± SD, *n* = 3, *p < 0.05; **p < 0.01; ***p < 0.001; ns, not significant, two‐tailed paired Student's *t* test. E,G) Data represent three independent experiments.

To understand the broader relevance of the DDIT3‐adaptive mechanism involving glutamine deprivation, we implemented a genetic approach that employed DDIT3 gene knockout in mice. CRISP‐CAS9 was used to eliminate DDIT3 expression by deleting the intronic region between exons 3 and 4 of *DDIT3* (**Figure** **5**A, upper) with homozygous WT and DDIT3‐KO animals distinguished using a dual PCR reaction‐based genotyping strategy (Figure 5A, lower). To then examine the impact of DDIT3 on body growth, WT or DDIT3 KO mice were raised on either standard chow or fed continuously with a glutamine deficient diet. Irrespective of the presence or absence of DDIT3, mice maintained without glutamine were smaller in weight, with notably larger spleens and yellowish livers while other organs were not noticeably affected (Figure 5B). Interestingly, analysis of plasma lactate levels showed higher levels in glutamine deprived mice suggesting increased glycolysis (Figure 5C). However, DDIT3 KO mice exhibited an overall reduction in lactate levels compared with WT mice indicating DDIT3 expression generally affected glycolysis. On this basis, we then evaluated the metabolic profiles of these mice using GTT (glucose tolerance test) in ITT (insulin tolerance test) assays where WT and DDIT3‐KO mice were fed on standard chow or the glutamine deficient diet. After glucose challenge, the blood glucose spike of DDIT3‐KO mice on the glutamine restriction diet was much higher than WT mice or DDIT3‐KO mice on the normal diet (Figure 5D, left). Similarly, for the ITT assay, the blood glucose levels of DDIT3‐KO mice were higher than WT controls, with mice fed the glutamine deficient diet showing elevated level when compared with the other three groups (Figure 5D, right). These findings indicate the DDIT3‐KO mice, particularly when maintained on a glutamine deficient diet, show metabolic syndrome phenotypes with hyperglycemia or diabetes along with insulin resistance.

**Figure 6:**
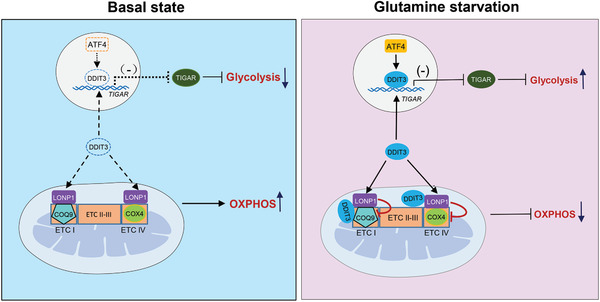
Working model for the regulation of DDIT3 on glycolysis and mitochondrial respiration.

Since the spleen and liver were the organs majorly affected by glutamine deprivation, we surveyed these tissues for changes in the expression of DDIT3, TIGAR, and the DDIT3‐associated respiratory components. Consistent with the in vitro data, mice fed with diet without glutamine exhibited increased DDIT3 protein expression in WT spleen and liver tissues although no expression was detected in the KO mice (Figure 5E). Notably, there were concordant reductions in TIGAR expression in glutamine‐deprived tissues in WT mice but not in DDIT3 KO mice. Moreover, the decreased TIGAR protein levels were correlated with reductions at the mRNA level (Figure 5F). Turning to examine DDIT3‐associated mitochondrial components revealed there were decreases in COQ9 and COX4 expression in WT but not DDIT3 KO mice spleen and liver accompanying glutamine deprivation (Figure 5G). Instructively, glutamine deprivation induced LONP1 in both WT and DDIT3 KO mice but this increase was not associated with the decreased expression of either COQ9 or COX4. Collectively these data support the mechanistic actions of DDIT3 disclosed in the preceding in vitro experiments and further suggest that DDIT3 enacts the same physiological role in vivo.

Lastly, since many of the mechanistic experiments conducted were performed in the context of a human cancer cell line, we evaluated the growth of WT and DDIT3‐KO HepG2 as xenografts in nude mice fed either a normal or glutamine‐free diet. Assessing growth after 4 weeks showed, as expected, the growth of WT tumors was inhibited when mice were fed the glutamine‐free diet. Moreover, the tumors from DDIT3‐KO cells were significantly smaller than WT groups fed normal or glutamine‐free diets (Figure 5H). These results imply that DDIT3 promotes tumorigenesis in vivo, assisting tumor cells to survive metabolic stress such as glutamine starvation (**Figure** **6**).

## 3 Discussion

DDIT3 is a key stress response regulator activated in diverse settings ranging from DNA damage, ER stress, hypoxia, and nutrient deprivation.^[^ ^32^ ^]^ DDIT3 protein accumulates under these conditions through multifaceted effects on transcription, mRNA stability, and translation.^[^ ^33^ ^]^ Herein, we establish that DDIT3 induction is involved in moderating the metabolic reprogramming response of cancer cells in response to limited glutamine availability. DDIT3 was regulated through the GCN2‐ATF4 axis which has been associated with amino acid deprivation responses which contrasts the classical role of DDIT3 in ER stress responses involving the PERK‐eIF2*α*‐ATF4 axis.^[^ ^38^ ^]^ Two complementary mechanisms involving DDIT3 were disclosed, acted out in discrete subcellular locations. The first, perhaps unsurprisingly given its canonical role as a transcription factor, was the nuclear role of DDIT3 in mediating activation of glycolysis through suppression of TIGAR. Second, noting reports that DDIT3 can also be expressed in the cytoplasm,^[^ ^51^, ^52^ ^]^ we discovered a pool of mitochondrial localized DDIT3 that functions to suppress OXPHOS. The summary actions of DDIT3 were shown to benefit the long‐term higher survival of cancer cells when exogenous glutamine is limiting.

Cancer cells are well known to switch their metabolism toward aerobic glycolysis even though this is not an efficient approach for ATP production. The advantages conferred have been endlessly debated and are unlikely to have a single explanation. Most prominently this feature has been associated with adaptation to hypoxic conditions^[^ ^26^ ^]^ although the highly glycolytic phenotype of cancer cells may be evident well before hypoxia develops in the tumor microenvironment (TME).^[^ ^53^ ^]^ Indeed, increased glycolysis itself is instrumental in establishing TME characteristics such as acidosis which results from the export of its end‐products lactate and H^+^ ions. It is known that aerobic glycolysis is impacted by glutamine deprivation stress^[^ ^28^, ^29^ ^]^ where enhanced glycolytic flux can increase the expression of glycolytic enzymes.^[^ ^30^ ^]^ However, the actions of DDIT3 were not leveled at individual glycolytic enzymes, rather indirectly through transcriptional inhibition of the negative glycolytic pathway regulator, TIGAR. Thus, activation of glycolysis through DDIT3 during chronic glutamine shortages appears to represent an economical means to maintain ATP levels and supporting long‐term cancer cell survival. Intriguingly, although TIGAR is a target of p53, the DDIT3‐mediated suppression of TIGAR occurred in a p53‐independent manner. Since p53 function is lost in over 50% of tumors, and both DDIT3 and TIGAR mutations are comparatively rare,^[^ ^54^ ^]^ this suggests that the DDIT3‐TIGAR‐glycolytic axis would remain intact in the majority of cancer cells.

Previous studies have shown also that accentuated aerobic glycolysis often accompanies reduced mitochondrial OXPHOS in order to maintain energetic balances.^[^ ^27^ ^]^ It is important to note that switch away from the TCA cycle does not mean it ceases to function since intermediates from glycolysis and the TCA cycle are both needed to support macromolecule biosynthesis. Nevertheless, a clear point of relevance regarding glutamine metabolism is the intrinsic role of OXPHOS in ROS production. Maintaining redox homeostasis is a delicate balance since although high ROS levels are cytotoxic because of oxidative stress, cancer cells also derive benefits from sustaining higher than normal levels of ROS which promotes by activation of pro‐oncogenic signaling pathways.^[^ ^55^ ^]^ Instructively, reductions in ROS resulting from decreased OXPHOS activity can inhibit cancer cell growth.^[^ ^56^ ^]^ In this respect, the mitochondria‐localized pool of DDIT3 makes a significant impact in moderating the temporal ROS responses during glutamine deprivation.

The initial response to glutamine deprivation evident at 4 h was the increased levels of cellular ROS which then gradually diminished over 48 h to be significantly decreased compared to basal levels. Consistently, OCR measurements demonstrated reduced OXPHOS activity at 48 h post‐withdrawal of glutamine. However, although the same trend of reduced OCR was evident in DDIT3‐KO cells, the OCR levels were increased in comparison to their WT equivalents. ROS levels still decreased in DDIT3‐KO cells over the time course, but it was clear that DDIT3 expression acted to dampen high ROS levels under glutamine deprivation conditions. The main sites of ROS generation are ETC respiratory Complexes I, II, and III with DDIT3 specifically shown to post‐translationally impair the levels of subunits COQ9 of Complex I and COX4 of Complex IV. Certainly, there is an increasing focus on the roles of mitochondrial proteases in controlling the balanced expression of mitochondrial proteins.^[^ ^50^ ^]^ It is pertinent to note that DDIT3 did not affect Complex II which closely connects the ETC with the TCA cycle through SDHA (Succinate Dehydrogenase Complex Flavoprotein Subunit A), which may help maintain normal function of TCA cycle.^[^ ^57^ ^]^ Nevertheless, further details of the DDIT3‐mediated perturbation of the ETC in mitochondria under glutamine deprivation, and perhaps other stress conditions, still require further investigating.

A further notable point of difference can be seen in comparing the actions of DDIT3 against COQ9 and Complex I versus COX4. Apart from the patently reduced expression of COQ9, there were general reductions in the levels of all other Complex I components, namely, NDUFS1 and to a lesser extent ETFDH, ETFDA, and NDUFB5 (Figure 3E). How this occurs is not clear, but one possibility is a domino effect where destabilizing one complex component may lead to reductions in other components. In contrast, among Complex IV only COX4 levels were noticeably affected. We found that the expression of LONP1, among other mitochondrial proteases, specifically increased with glutamine deprivation, where it is responsible for hydrolysis of both COQ9 and COX4. Interestingly, LONP1 was shown to promote COX4‐1 isoform degradation under hypoxia^[^ ^58^ ^]^ but whether DDIT3 could be involved is not clear. One salient difference is that DDIT3 directly binds to COQ9 and facilitates a complex with LONP1 whereas DDIT3 does not bind to COX4, although it remains necessary for its degradation by LONP1.

A final question is how DDIT3 is recruited to mitochondria? Mitoribosomes manufacture much of the protein content of mitochondria^[^ ^59^ ^]^ but numerous extraneous proteins are also imported to regulate mitochondrial function. This can be aided through the TOM complex (TOM20, TOM70, and TOM40), TIM complex (TIM21, TIM22, and TIM23), or cytosolic chaperones proteins such as Hsp70 and Hsp90.^[^ ^60^ ^]^ Alternatively, cytosolic precursors of mitochondria‐targeted proteins possess matrix‐targeting sequences (MTSs) at their N‐terminus. MTSs consist of hydrophobic and positively charged residues, these 10–80 amino acid stretches serve to integrate with membranes.^[^ ^60^ ^]^ Nevertheless, MTSs are not obligatory, e.g., Bcl‐2 or Fis1 which both lack an N‐terminal pre‐sequence are still able to translocate into mitochondria.^[^ ^61^ ^]^ However, bioinformatics predictions indicate that DDIT3 has a very low likelihood of containing a functional mitochondrial transfer peptide. We therefore assume DDIT3 translocation requires aiding factors as occurs with APE1 that also lacks a conventional MTS but translocates to the mitochondria inner membrane via interacting with Mia40, a component of the mitochondrial import machinery.^[^ ^62^ ^]^

## 4 Experimental Section

##### Cell Culture

Cell lines (293T, MCF7, HCT116 (p53^+/+^), and HepG2) were cultured in high glucose (25 × 10^−3^ m) Dulbecco's modified Eagle's medium (Thermo Fisher Scientific) with 10% v/v fetal bovine serum (BI, Biological Industries), 4 × 10^−3^ m L‐glutamine, 1% v/v penicillin‐streptomycin (Gibco), and 1 × 10^−3^ m pyruvate (Gibco) and maintained at 37 °C in a humidified 5% CO_2_‐containing atmosphere. Cell line authenticity was verified by short tandem repeat analysis.

##### Microarray Analysis

Transcriptomic analyses were obtained on a contract basis BGI (The Beijing Genomics Institute) Company, Shenzhen, China with data analyses performed according to publically available instructions (www.bgionline.cn).

##### RNA Inference and Transfections

Gene knockdown experiments were conducted by lentiviral‐mediated transduction with short hairpin RNAs (shRNAs). Lentiviral particles were generated by transfection of 293T cells with PLKO.1 vectors containing specific shRNAs (Table S2, Supporting Information) along with pREV, pGag, pVSVG at the ratio of 2:2:2:1 in Opti‐MEM medium (Gibco) for 48 h. Supernatants were filtered with 0.45 µm filter before cells infection, added to target cells for 24 h before selection with 5 µg mL^−1^ puromycin. Alternatively, transfections were performed with the indicated plasmids (Table S3, Supporting Information) using the Lipofectamine‐2000 reagent (Invitrogen) according the manufacturer's instructions.

##### Glycolysis and Mitochondrial Stress Tests

Assays were performed using the Seahorse XFe96 analyzer (Agilent) according to the manufacturer's instructions. Cells were seeded at 1×10^4^ cells per well in 96‐well XF cell culture micro‐plates for 24 h before performing glycolysis stress tests at 37 °C in XF base medium (2 × 10^−3^ m glutamine, pH 7.4) with sequential additions of glucose (10 × 10^−3^ m), oligomycin (1 × 10^−6^ m), and 2‐DG (5 × 10^−3^ m). Alternatively, mitochondria stress tests were performed in XF base medium (1 × 10^−3^ m pyruvate, 2 × 10^−3^ m glutamine, 10 × 10^−3^ m glucose, pH 7.4) with sequential additions of oligomycin (1.0 × 10^−6^ m), FCCP (0.25 × 10^−6^ m), and Rot/AA (0.5 × 10^−6^ m). Data were analyzed by the Seahorse XF Glycolysis Stress Test and Mitochondria Stress Test Report Generator packages, respectively.

##### Subcellular Fractionation

For cytosol/nuclear fractions, cell suspensions were incubated with hypotonic buffer (25 × 10^−3^ m Tris‐HCl pH 7.4, 1 × 10^−3^ m MgCl_2_, 5 × 10^−3^ m KCl) on ice for 5 min before adding an equal volume of hypotonic buffer containing 1% NP‐40 for another 5 min. Homogenates prepared by pipetting were then centrifuged at 5000 x *g* for 10 min at 4 °C, and the supernatant collected as the cytosol fraction. Pellets were rinsed twice with hypotonic buffer and re‐suspended in nuclear resuspension buffer (20 × 10^−3^ m 4‐(2‐hydroxyethyl)‐1‐piperazineethanesulfonic acid pH 7.9, 400 × 10^−3^ m NaCl, 1 × 10^−3^ m ethylenediaminetetraacetic acid, 1 × 10^−3^ m ethylene glycol‐bis(*β*‐aminoethyl ether)‐*N*,*N*,*N*′,*N*′‐tetraacetic acid, 1 × 10^−3^ m dithiothreitol, 1 × 10^−3^ m phenylmethylsulfonyl fluoride). After incubation on ice for 30 min, the samples were centrifuged at 12 000 x *g* for 10 min at 4 °C and the supernatant collected as the nuclear fraction. Similarly, cytosolic/mitochondrial were performed with the mitochondria isolation kit (Sigma) but with stepwise centrifugation of homogenates at 600 x *g* for 10 min, and then at 11 000 x *g* for 10 min to derive supernatant (cytosol fraction) and pellet fractions, the latter re‐suspended with mitochondria storage buffer as the mitochondrial fraction.

##### Western Blotting and Immunoprecipitation

Cell lysates were prepared with radio immunoprecipitation assay buffer containing protease inhibitors (Beyotime) and clarified by centrifugation at 10 000 x *g* for 15 min at 4 °C. Equal amounts as determined using the Bio‐Rad RC/DC protein assay were electrophoresed by sodium dodecyl sulfate‐polyacrylamide gel electrophoresis and transferred to nitrocellulose membranes. Membranes were blocked with 4% skim milk and incubated with primary antibodies overnight at 4 °C, decorated with horseradish peroxidase‐conjugated secondary antibodies (Table S5, Supporting Information) with detection using chemiluminescence (Advansta). Alternatively, for immunoprecipitations, cell lysates prepared with IP buffer (0.5% NP‐40, 20 × 10^−3^ m Tris pH 7.4, 150 × 10^−3^ m NaCl, 1.5 × 10^−3^ m MgCl_2_ and protease inhibitor cocktail (Solarbio)) were incubated with primary antibodies adsorbed to protein A/G‐sepharose (Invitrogen) beads for 4 h, washed five times with IP buffer. Antibody sources/dilutions are shown in Table S4 with uncropped immunoblot scans provided in the Supporting Information.

##### Nile Red Staining

Cells grown on glass coverslips were rinsed with warmed phosphate‐buffered saline (PBS) and fixed in 4% formaldehyde before staining with a solution containing Nile Red (8 µg mL^−1^) and Hoechst 33342 (0.1 µg mL^−1^) for 10 min at room temperature. After mounting the coverslips, images were collected using a ZEISS LSM 700 confocal microscope. For quantitation, cells were harvested and cell suspensions were incubated with Nile Red solution, washed with PBS, and transferred to 96‐well plate before measuring the absorbance at OD 530 nm.

##### Immunofluorescence Staining

Cells grown on glass coverslips were fixed in 4% formaldehyde for 15 min at room temperature before permeabilization (0.2% Triton X‐100) for 10 min at room temperature. Samples were blocked (1% bovine serum albumin) for 60 min at room temperature and washed using PBS with 0.05% Tween‐20 (PBST) before the addition of primary antibodies in PBST overnight at 4 °C. Samples were washed and bound antibodies decorated with appropriate fluorochrome‐conjugated secondary antibodies diluted in PBST for 1 h at room temperature. Samples were counterstained with Hoechst 33342, mounted in anti‐fade mounting medium, and images were collected using a ZEISS LSM 700 confocal microscope.

##### CRISPR/Cas9

CRISPR/Cas9‐mediated DDIT3, TIGAR, and ATF4gene‐editing vectors were constructed by annealing gRNA oligonucleotide pairs (Table S3, Supporting Information) and subcloning into lentiCRISPRv2 (one vector system) according to the Zhang laboratory protocol. Lentiviral particles produced as described above using a 1:2:2 mixture of plasmids (Pmd2.g, PSPAX2 and lentiCRISPRv2) were used to transduce target cells. After selection with 5 µg mL^−1^ puromycin, stably infected cells were plated in 96‐well plates and single cell clones were screened by Western blot and gDNA sequencing to obtain DDIT3, TIGAR, and ATF4 knockout cells.

##### qRT‐PCR

RNA extraction using the TRIzol Reagent (Invitrogen) was performed on cells grown in 12‐well plates. cDNA was prepared from total RNA using the PrimeScriptTM RT reagent kit (TaKaRa) according to the manufacturer's instructions. Quantitative RT‐PCR analyses were performed using the specified primers (Table S4, Supporting Information) with the One‐Step PrimeScript RT‐PCR kit (TaKaRa). Relative expression values were calculated using the comparative *C_t_* method normalized against the beta‐microglobulin housekeeping gene.

##### Metabolite Assays

Cellular ATP, ROS, extracellular lactate levels, mitochondrial ROS, and glucose uptake were measured using the ATP assay Kit (Biovision), ROS assay kit (Beyotime), Lactate production assay kit (Biovision), Mitochondrial ROS Detection Assay kit (Cayman Chemical), and 2‐NBDG glucose uptake assay kit (Abcam), respectively, according to the manufacturer's instructions. Fru‐2,6‐BP levels in HepG2 DDIT3‐WT and ‐KO cells treated with or without glutamine were determined by Quantitative Metabolomics based on LC‐MS from BGI (The Beijing Genomics Institute) company, Shenzhen, China.

##### Luciferase Reporter Assays

Cells seeded in 24‐well plates were co‐transfected with the indicated pGL3‐based reporter plasmids (Table S3, Supporting Information) along with Renilla luciferase. After 24 h, the results were assessed using the Dual‐Luciferase Reporter Assay System (Promega) with firefly luciferase values corrected against Renilla measurements according to the manufacturer's instructions.

##### Chromatin Immunoprecipitation

ChIP assays were performed with the Millipore ChIP kit according to the manufacturer's instructions. Bound DNA fragments were subjected to semi‐quantitative RT‐PCR using the specified primers (Table S4, Supporting Information).

##### DDIT3 Knockout and Animal Experiments

A general chromosome‐engineering technique was used to target the imprinted *DDIT3* box gene cluster by deleting an intronic fragment between exons 3 and 4 using CRISPR‐CAS9 (Figure 5A, upper). After derivation, *DDIT3+/−* C57/BL mice were maintained under SPF conditions under contract with Cyagen Biology Company. Following genotyping, backcrossed mice were divided into *DDIT3*+/+ and *DDIT3*−/− groups (Table S4, Supporting Information and Figure 5A, upper) with half of each group, then fed a standard diet or glutamine deprivation diet (Medicience Ltd.). Following mating, 3 weeks old progenies were weaned and these mice were maintained for a further 9 weeks on the specified diets.

##### Xenograft Model

BALB/c nude mice (5 weeks old, male) were obtained from Shanghai SLAC Laboratory Animal Co. Ltd. After 3 days acclimatization, mice were injected subcutaneously (s.c.) with 5.0 × 10^6^ WT and DDIT3‐KO HepG2 cells on alternate sides of each mouse. Mice were randomized into normal diet and glutamine‐free diet groups and maintained for 4 weeks before the animals were humanely sacrificed and the tumors were excised and weighed. Studies were conducted with the approval from the Animal Research Ethics Committee of University of Science and Technology of China.

##### GTT and ITT

Mice were fasted overnight for 12 h, and fasting blood glucose levels were determined by glucometer using tail vein blood. For GTT, mice were then injected i.p. with 10 uL of glucose solution (200 mg mL^−1^) per gram of body weight and blood glucose was measured at 15, 30, 60, 90, and 120 min post injection. For ITT, fasted mice were injected i.p. with insulin at 0.75 U kg^−1^ body weight using a stock insulin solution of 0.1 U mL^−1^ in 0.9% NaCl. Blood glucose was measured at 15, 30, 45, and 60 min after insulin injection. Studies were conducted with approval from the Animal Research Ethics Committee of University of Science and Technology of China.

##### Statistical Analysis

Data were assumed to be normally distributed and continuous variables expressed as mean ± SD. All analyses were performed by two‐tailed Student's *t*‐test using GraphPad Prism 8 with significance defined as *p* ≤ 0.05. Reproducibility and the number of replicates used are defined in the corresponding figure legends.

## Conflict of Interest

The authors declare no conflict of interest.

## Author Contributions

M.L., R.F.T., Q.Y.Z., M.W., and L.X.L. designed the research. M.Y.L. performed the most experiments. R.H.S. helped with Seahorse analysis. X.D.Z., J.M.L., and J.T.L. carried out data analysis. R.F.T, M.W., and M.Y.L. wrote the manuscript.

## Supporting information

Supporting Information

## Data Availability

The data that support the findings of this study are available from the corresponding authors upon reasonable request.
