# Supplementary material for: DDIT3 Directs a Dual Mechanism to Balance Glycolysis and Oxidative Phosphorylation during Glutamine Deprivation
Source: Adv Sci (Weinh). 2021 Mar 27;8(11):2003732. doi: 10.1002/advs.202003732 (PMC8188220; doi:10.1002/advs.202003732)
Supplement: Supplementary file 1 — Supporting Information [file ADVS-8-2003732-s001.pdf]

© 2021 Wiley-VCH GmbH

ADVANCED SCIENCE
Open Access

Supporting Information

for *Adv. Sci.,* DOI: 10.1002/advs.202003732

DDIT3 Directs a Dual Mechanism to Balance Glycolysis and Oxidative Phosphorylation during Glutamine Deprivation

Mingyue Li, Rick Francis Thorne, Ronghua Shi, Xu Dong Zhang, Jingmin Li, Jingtong Li, Qingyuan Zhang,, Mian Wu,* and Lianxin Liu**

Figure S1. DDIT3 is up-regulated following glutamine deprivation and promotes cancer cell glycolysis

S1A

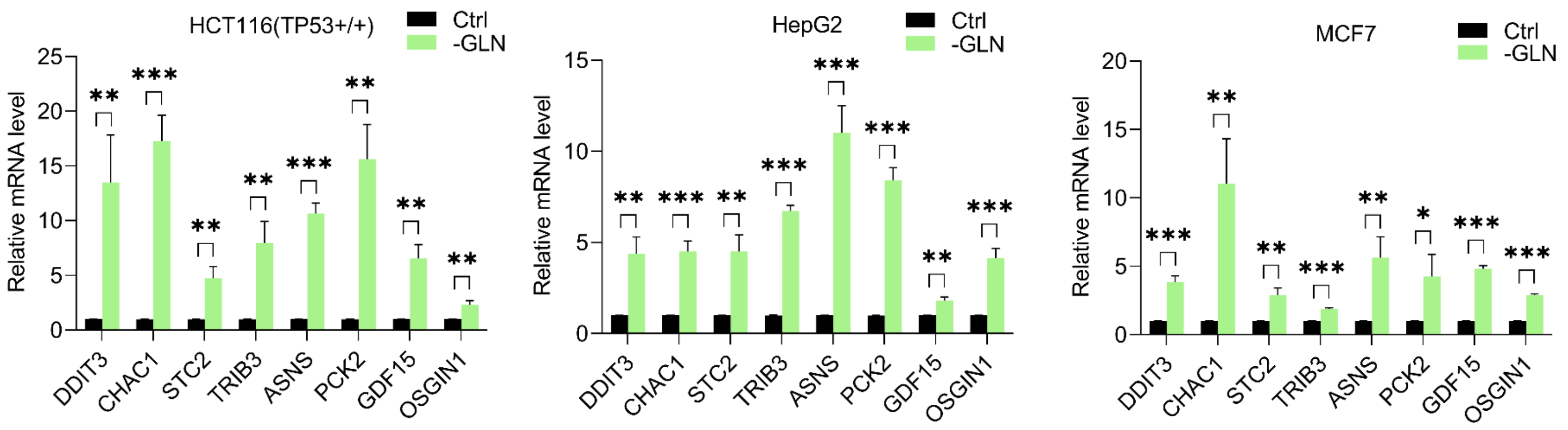

S1B

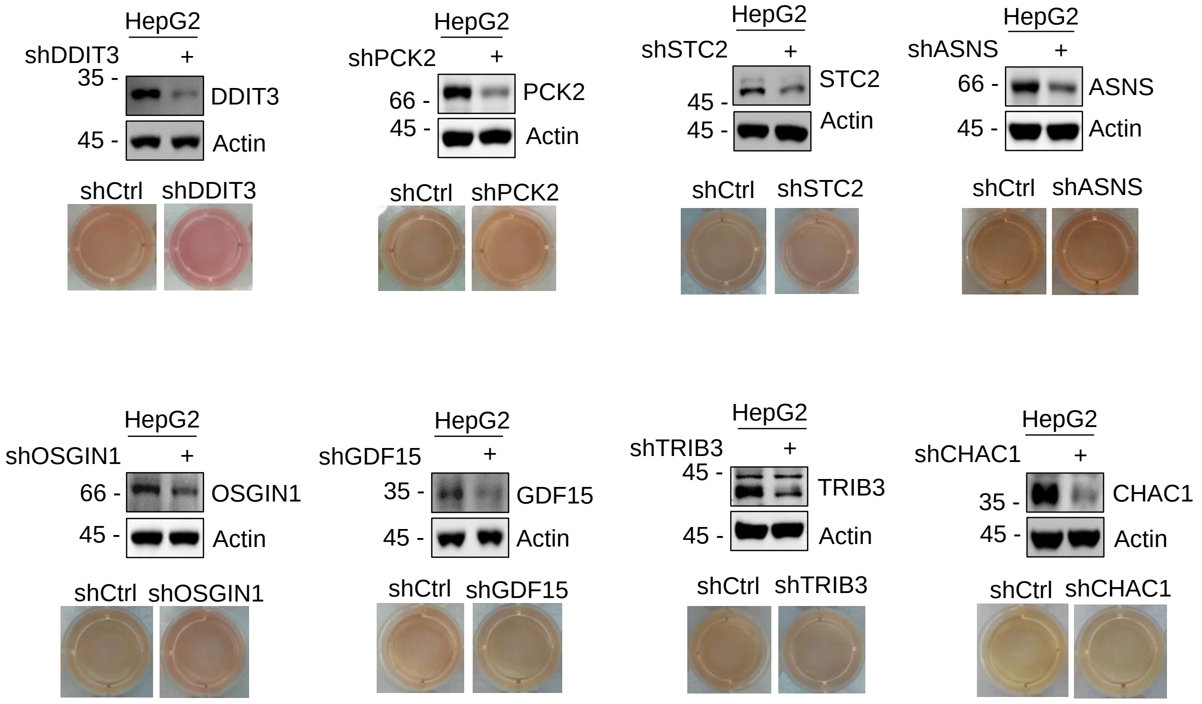

S1C

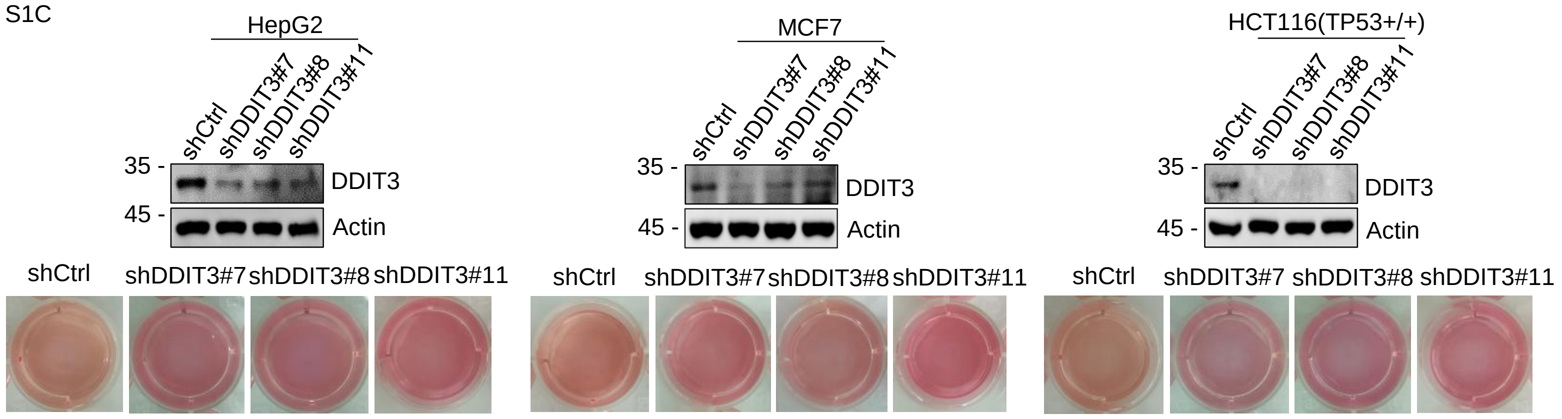

Figure S2. Identification of a GCN2/ATF4/DDIT3 regulatory axis and the effects of glutamine deprivation on lipid metabolism

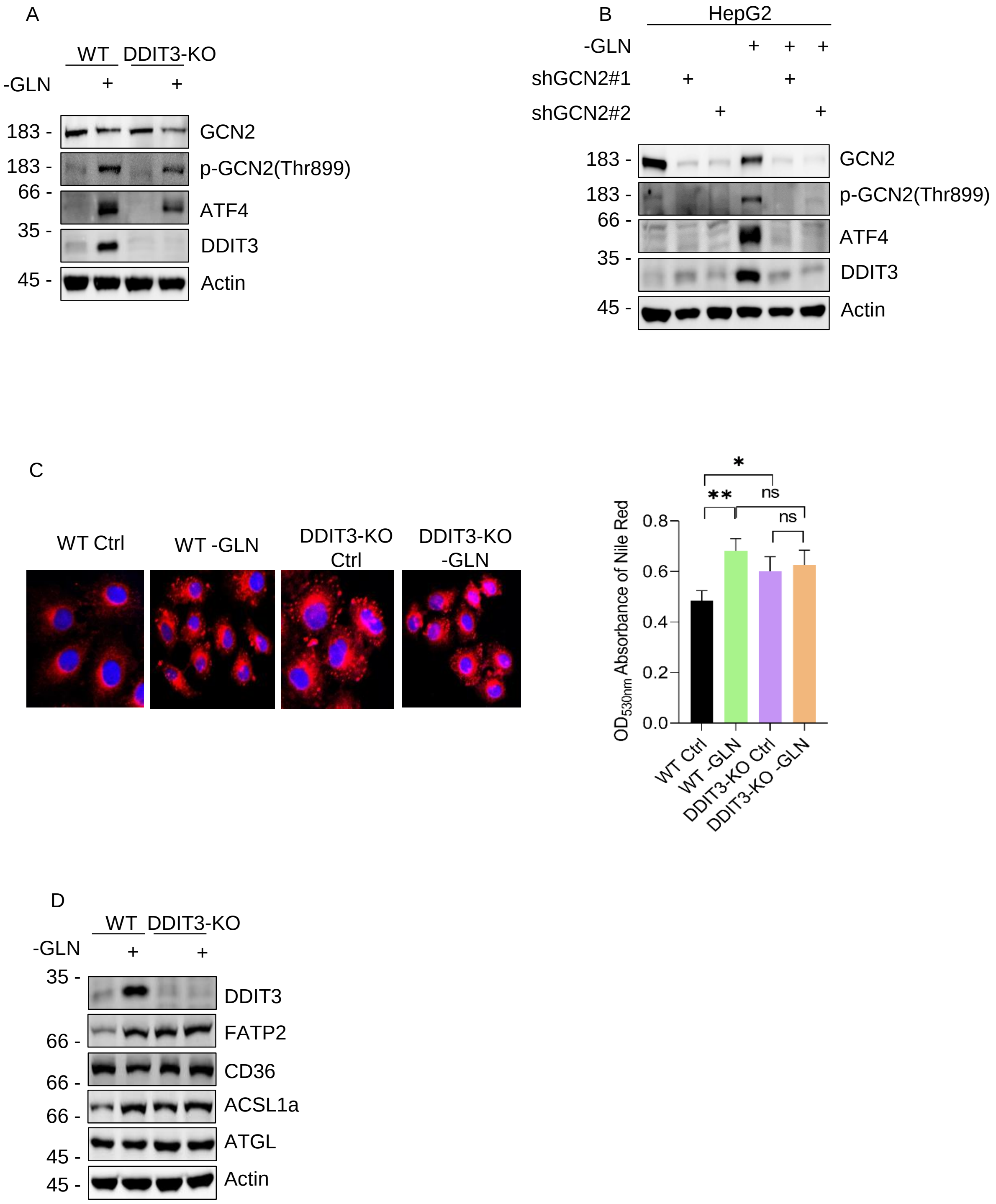

Table S1. Microarray Analyses of Transcriptomes Information

Gene ID	Ctrl FPKM	Treat FPKM	log2 (Treat/Ctrl)	Qvalue (Ctrl-vs-Treat)	Pvalue (Ctrl-vs-Treat)
KLHDC7B	0	3.333	9.995264	2.99E-87	1.04E-87
MTPN	0	1.533	9.25186	2.16E-58	1.04E-58
FGF21	0	2.92	8.607623	5.47E-41	3.40E-41
NARR	0	3.593	8.214456	6.08E-33	4.33E-33
PLGLB2	0	0.4	6.929887	5.52E-16	5.86E-16
LOC107987425	0	0.413	6.879037	1.57E-15	1.69E-15
FSBP	0	0.163	6.522522	1.08E-12	1.27E-12
IGFN1	0.02	1.623	6.460281	NA	NA
CSTA	0	0.85	6.130576	3.53E-10	4.58E-10
LOC102724995	0	0.24	5.947923	3.45E-09	4.66E-09
CORO7-PAM16	0	0.15	5.828975	1.34E-08	1.86E-08
STC2	1.216	41.39	5.087083	0	0
CFAP161	0	0.203	5.008585	1.69E-05	2.76E-05
ADM2	0.526	14.916	4.830778	0	0
DRD5	0	0.116	4.828013	5.35E-05	8.98E-05
LOC107985734	0	0.253	4.813514	5.83E-05	9.81E-05
CTAGE15	0	0.1	4.716058	0.000102	0.000175
LRMP	0	0.113	4.621562	0.000171	0.000297
FAM47E-STBD1	0	0.073	4.490371	0.000333	0.000591
CHAC1	1.333	35.813	4.465105	0	0
TBC1D3K	0	0.06	4.401388	0.000507	0.000913
HOXB9	0.013	0.3	4.364398	5.09E-11	6.38E-11

TAS2R31	0.016	0.233	3.884597	0.000398	0.000712
INHBE	0.943	12.726	3.752901	0	0
MLXIPL	0.063	0.79	3.745725	1.31E-28	1.02E-28
WNK4	0.11	1.566	3.741857	7.08E-65	3.14E-65
ANK2	0.01	0.093	3.658088	2.19E-09	2.93E-09
MIOX	0.09	1.426	3.603447	4.03E-38	2.62E-38
CYP4A11	0.016	0.1	3.518058	0.000713	0.001297
ASNS	19.553	206.056	3.511509	0	0
GNG13	0.02	0.23	3.506085	0.000756	0.001379
PHGDH	19.25	212.75	3.466134	0	0
FREM1	0.003	0.023	3.358528	0.000591	0.001068
QRICH2	0.08	0.7	3.288917	3.83E-45	2.24E-45
SLC5A2	0.033	0.38	3.273837	6.02E-10	7.89E-10
GPR35	0.03	0.253	3.243051	6.45E-07	9.74E-07
NUPR1	37.016	347.393	3.228793	0	0
SCT	0.09	0.806	3.194141	1.13E-06	1.73E-06
UNC5B	2.2	19.713	3.166267	0	0
FLRT1	0.073	0.64	3.133772	2.63E-22	2.37E-22
OVOS2	0.053	0.44	3.125919	1.46E-19	1.41E-19
DDR2	0.206	1.826	3.121656	1.52E-89	5.16E-90
TGIF2-C20orf24	0.106	0.883	3.041739	4.98E-10	6.51E-10
SLC4A10	0.006	0.06	3.008585	0.000211	0.000369
SLAMF9	0.013	0.113	3.008585	0.000211	0.000369
S100P	8.646	69.486	3.005767	NA	NA
ULBP1	0.17	1.27	2.978002	3.38E-36	2.26E-36
DGAT2	0.336	2.62	2.960175	1.79E-55	8.98E-56
SLC7A11	4.026	30.803	2.925094	0	0

GZMM	0.043	0.32	2.921123	0.00037	0.00066
PLAC1	0.133	0.973	2.921123	1.46E-10	1.87E-10
ECM2	0.31	2.346	2.921123	6.20E-66	2.71E-66
SULT4A1	0.02	0.156	2.921123	0.00037	0.00066
AKNA	0.11	0.816	2.908632	8.28E-38	5.41E-38
CREB5	0.033	0.18	2.862932	2.11E-12	2.52E-12
TRIB3	25.683	191.246	2.857759	0	0
CILP2	0.063	0.436	2.850039	5.66E-17	5.85E-17
SLC7A5	228.366	1599.773	2.839095	0	0
SESN2	4.71	33.443	2.827562	0	0
PPAN-P2RY11	1.836	12.903	2.825458	0	0
TAS2R19	0.06	0.416	2.795592	5.58E-05	9.38E-05
FBXW10	0.04	0.243	2.790945	1.83E-07	2.68E-07
FAM129A	3.413	23.383	2.776269	0	0
DDIT3	6.043	45.743	2.729848	0	0
CLDN9	0.05	0.306	2.728477	7.55E-07	1.15E-06
GNG3	0.326	2.113	2.716247	3.24E-17	3.32E-17
TSSK2	0.073	0.473	2.681866	2.12E-08	2.96E-08
SLC3A2	158.12	1046.54	2.642476	0	0
HLF	0.016	0.106	2.599194	3.72E-06	5.85E-06
PCK2	9.266	55.286	2.5879	0	0
RASGRP1	0.013	0.076	2.584088	0.000284	0.0005
OSGIN1	3.466	20.616	2.57253	NA	NA
FAM43A	0.076	0.44	2.564979	4.71E-12	5.69E-12
CCL17	0.1	0.56	2.457175	1.78E-05	2.92E-05
GPR1	1.043	5.623	2.448136	6.74E-86	2.37E-86
CD177	0.323	1.833	2.444685	1.06E-25	8.82E-26

APOE	0.116	0.636	2.435696	6.86E-07	1.04E-06
LOC728715	1.976	10.63	2.427389	5.43E-42	3.33E-42
CDHR5	0.026	0.11	2.423623	0.000819	0.001498
CRHR2	0.023	0.123	2.423623	0.000819	0.001498
P2RY11	1.203	6.416	2.412425	1.90E-88	6.52E-89
CLGN	1.3	6.883	2.407119	NA	NA
C19orf71	3.67	19.873	2.380554	6.81E-85	2.42E-85
BGLAP	0.41	2.04	2.318791	1.42E-11	1.75E-11
ARHGAP25	0.046	0.153	2.31344	0.000336	0.000596
CCDC13	0.086	0.49	2.305786	2.02E-07	2.96E-07
CCDC17	0.19	0.873	2.293686	1.52E-15	1.63E-15
GDF15	44.313	214.65	2.275989	0	0
MSANTD1	0.05	0.083	2.26453	0.000479	0.000861

Table S2. Plasmid Information for Knockdown Genes

Name	Resistance marker	Vector
shCtrl	Ampicillin	pLKO.1
shATF4(#5)	Ampicillin	pLKO.1
shDDIT3(#7)	Ampicillin	pLKO.1
shDDIT3(#8)	Ampicillin	pLKO.1
shDDIT3(#11)	Ampicillin	pLKO.1
shCHAC1(#3)	Ampicillin	pLKO.1
shSTC2(#7)	Ampicillin	pLKO.1
shASNS(#6)	Ampicillin	pLKO.1
shOSGIN1(#11)	Ampicillin	pLKO.1
shGDF15(#11)	Ampicillin	pLKO.1
shTRIB3(#11)	Ampicillin	pLKO.1

shPCK2(#8)	Ampicillin	pLKO.1
shLONP1(#2)	Ampicillin	pLKO.1
shCOQ9(#10)	Ampicillin	pLKO.1
shETFA(#8)	Ampicillin	pLKO.1
shTIGAR(#4)	Ampicillin	pLKO.1
shGCN2(#1)	Ampicillin	pLKO.1
shGCN2(#2)	Ampicillin	pLKO.1
lentiCRISPR V2	Ampicillin	lentiCRISPR V2
CRISPR-Cas9-KO DDIT3(clone2-1)	Ampicillin	lentiCRISPR V2
CRISPR-Cas9-KO TIGAR(clone2-8)	Ampicillin	lentiCRISPR V2
CRISPR-Cas9-KO ATF4(clone1-2)	Ampicillin	lentiCRISPR V2
Rev	Ampicillin	Rev
Gag	Ampicillin	Gag
VSV-G	Ampicillin	VSV-G
psPAX2	Ampicillin	psPAX2
pMD2.G	Ampicillin	pMD2.G

Table S3. Plasmid Information for Transfection Genes

Name	Resistance marker	Vector
pGL3	Ampicillin	pGL3-Basic
pGL3-Site1	Ampicillin	pGL3-Basic
pGL3-Site2	Ampicillin	pGL3-Basic
pGL3-Site3	Ampicillin	pGL3-Basic
pGL3-Site3-mut	Ampicillin	pGL3-Basic

Flag	Ampicillin	pSin-3Xflag
Flag-DDIT3	Ampicillin	pSin-3Xflag
Flag-COQ9	Ampicillin	pSin-3Xflag
Flag-COX4	Ampicillin	pSin-3Xflag
HA	Ampicillin	pCMV-HA
HA-LONP1	Ampicillin	pCMV-HA

Table S4. Primer Information

Usage	Name	Primer Sequence 5'-3'
Knockdown genes	shATF4(#5)	CCGGGCCTAGGTCTCTTAGATGATTCTCG AGAATCATCTAAGAGACCTAGGCTTTTT
Knockdown genes	shDDIT3(#7)	CCGGGCCAATGATGTGACCCTCAATCTCG AGATTGAGGGTCACATCATTGGCTTTTT
Knockdown genes	shDDIT3(#8)	CCGGCCTGGAAATGAAGAGGAAGAACTC GAGTTCTTCCTCTTCATTTCCAGGTTTTT
Knockdown genes	shDDIT3(#11)	CCGGGAACAGGAGAATGAAAGGAAACTC GAGTTTCCTTTCATTCTCCTGTTCTTTTT
Knockdown genes	shCHAC1(#3)	CCGGCCTTGAATACTTGCTGCGTCTCTCG AGAGACGCAGCAAGTATTCAAGGTTTTTT G
Knockdown genes	shSTC2(#7)	CCGGGAAGACGAACAGTCTGAGTATCTC GAGATACTCAGACTGTTCGTCTTCTTTTTT G
Knockdown genes	shTRIB3(#11)	CCGGCCAGGTCCATACTCTAGGTTTCTCG AGAAACCTAGAGTATGGACCTGGTTTTTG
Knockdown genes	shOSGIN1(#11)	CCGGCTCACCTCATCAGAGTTGTTTCTCG AGAAACAACTCTGATGAGGTGAGTTTTTT

		G
Knockdown genes	shGDF15(#11)	CCGGGCTCCAGACCTATGATGACTTCTCG AGAAGTCATCATAGGTCTGGAGCTTTTTG
Knockdown genes	shASNS(#6)	CCGGGCTCTGTTACAATGGTGAAATCTCG AGATTTCACCATTGTAACAGAGCTTTTTG
Knockdown genes	shPCK2(#8)	CCGGGCACATCCCAACTCTCGATTTCTCG AGAAATCGAGAGTTGGGATGTGCTTTTTG
Knockdown genes	shCOQ9(#10)	CCGGCCGGGTTAATGATGCAATGAACTCG AGTTCATTGCATCATTAACCCGGTTTTTG
Knockdown genes	shETFA(#8)	CCGGGCTTGACCAGAAATTAACAAACTC GAGTTTGTTAATTTCTGGTCAAGCTTTTT G
Knockdown genes	shLONP1(#2)	CCGGCCAGTGTTTGAAGAAGACCAACTC GAGTTGGTCTTCTTCAAACACTGGTTTTT G
Knockdown genes	shTIGAR(#4)	CCGGGCATGGAGAAACAAGATTTAACTC GAGTTAAATCTTGTTTCTCCATGCTTTTTG
Knockdown genes	shGCN2(#1)	CCGGCCAGATGTAGTTCCTGAAATACTCG AGTATTTCAGGAACTACATCTGGTTTTTG
Knockdown genes	shGCN2(#2)	CCGGCCAAAGGTCTATCAAATGAAACTC GAGTTTCATTTGATAGACCTTTGGTTTTTG
CRISPR-Cas9 construction	DDIT3-oligo1-F	CACCGCTGAAGACAGGACCTCTTGC
CRISPR-Cas9 construction	DDIT3-oligo1-R	AAACGCAAGAGGTCCTGTCTTCAGC
CRISPR-Cas9 construction	DDIT3-oligo2-F	CACCGCCAGCTGGACAGTGTCCCGA

CRISPR-Cas9 construction	DDIT3-oligo2-R	AAACTCGGGACACTGTCCAGCTGGC
CRISPR-Cas9 construction	DDIT3-oligo3-F	CACCGTCTCTGCAGTTGGATCAGTC
CRISPR-Cas9 construction	DDIT3-oligo3-R	AAACGACTGATCCAACTGCAGAGAC
CRISPR-Cas9 construction	TIGAR-oligo1-F	CACCGCCTGTGTTTACACCGCCCGG
CRISPR-Cas9 construction	TIGAR-oligo1-R	AAACCCGGGCGGTGTAAACACAGGC
CRISPR-Cas9 construction	TIGAR-oligo2-F	CACCGTCCAGCGTCTCTCCTCCGGG
CRISPR-Cas9 construction	TIGAR-oligo2-R	AAACCCCGGAGGAGAGACGCTGGAC
CRISPR-Cas9 construction	ATF4-oligo1-F	CACCGAATGAGCTTCCTGAGCAGCG
CRISPR-Cas9 construction	ATF4-oligo1-R	AAACCGCTGCTCAGGAAGCTCATTC
CRISPR-Cas9 construction	ATF4-oligo2-F	CACCGATCACAAGTGTCATCCAACG
CRISPR-Cas9 construction	ATF4-oligo2-R	AAACCGTTGGATGACACTTGTGATC
QRT-PCR	QRT-ATF4-F	AGGTGTTCTCTGTGGGTCTG
QRT-PCR	QRT-ATF4-R	GTACCTAGTGGCTGCTGTCT
QRT-PCR	QRT-DDIT3-F	GCTGGAAAGCAGCGCATGAA
QRT-PCR	QRT-DDIT3-R	GCGAGTCGCCTCTACTTCCC
QRT-PCR	QRT-CHAC1-F	GCGTGGCATACCAAGTGCAA

QRT-PCR	QRT-CHAC1-R	AGGCCAATGCCTTCAGTGGT
QRT-PCR	QRT-STC2-F	GAAGCCTGTGCTCCATCTTG
QRT-PCR	QRT-STC2-R	CCTGGAGAGCTTGGTTCTGT
QRT-PCR	QRT-TRIB3-F	CTGGTACCCAGCTCCTCTAC
QRT-PCR	QRT-TRIB3-R	CAAAGCGACACAGCTTGAGA
QRT-PCR	QRT-ASNS-F	ATCACTGTCGGGATGTACCC
QRT-PCR	QRT-ASNS-R	GCAGCCAATCCTTCTGTCTG
QRT-PCR	QRT-PCK2-F	GCAAGACCAACCTGGCTATG
QRT-PCR	QRT-PCK2-R	CCCAAAGAAGCCGTTCTCAG
QRT-PCR	QRT-GDF15-F	TCCGAAGACTCCAGATTCCG
QRT-PCR	QRT-GDF15-R	ATCTTCCCAGCTCTGGTTGG
QRT-PCR	QRT-OSGIN1-F	GACTGGATGCAGAAGAAGCG
QRT-PCR	QRT-OSGIN1-R	CTACAGCACCGGACACAAAG
QRT-PCR	QRT-TIGAR- F	TCCCAAGGATCTCCAAGCAA
QRT-PCR	QRT-TIGAR- R	AGCACCGTGACTCACAACTA
QRT-PCR	QRT-LONP1-F	AAAGCCCTGACTGCAGAGAT
QRT-PCR	QRT-LONP1-R	ATGTCGCTCAGGTAGATGGG
QRT-PCR	QRT-COQ9-F	GCAGAAGCTGGTACAGTTGG
QRT-PCR	QRT-COQ9-R	GCCAGTGCTCAATGTATGGG
QRT-PCR	QRT-COX4-F	GGAGAGCTTTGCTGAGATGAA
QRT-PCR	QRT-COX4-R	AGTGCTTCTGCCACATGATAA
QRT-PCR	QRT-Actin-F	GACCTGACTGACTACCTCATGAAGAT
QRT-PCR	QRT-Actin-R	GTCACACTTCATGATGGAGTTGAAGG
QRT-PCR	miceQRT-TIGAR-F	AATGTGCAGTTTACCCACGC
QRT-PCR	miceQRT-TIGAR-R	CTGGAGTCGTACTTCACCGC

QRT-PCR	miceQRT-Actin-F	AGGGAAATCGTGCGTGACAT
QRT-PCR	miceQRT-Actin-R	CTTCTCCAGGGAGGAAGAGGA
Plasmid construction	DDIT3-PCR-F	cgGAATTCATGGAGCTTGTTCCAGCCACTCCCC
Plasmid construction	DDIT3-PCR-R	ggACTAGTTCATGCTTGGTGCAGATTCACCATT
Plasmid construction	COX4-PCR-F	cgGAATTCATGTTGGCTACCAGGGTATTTAGCC
Plasmid construction	COX4-PCR-R	ggACTAGTTCACTTCTTCCACTCGTTCTTTTCG
Plasmid construction	COQ9-PCR-F	cgGAATTCATGGCGGCGGCGGCGGTATCTGGTG
Plasmid construction	COQ9-PCR-R	ggACTAGTTCACCGACGCTGGTTTAGACCTGTC
Plasmid construction	LONP1-insert1-F	tggccatggaggcccgaattcgcATGGCGGCGAGCACTGGC
Plasmid construction	LONP1-insert1-R	gtagatggggTTGTCCACCACCCGCTGG
Plasmid construction	LONP1-insert2-F	tggtggacaaCCCCATCTACCTGAGCGACA
Plasmid construction	LONP1-insert2-R	aacagcaccttggaCAAGTCCACGGGCACGTCC
Plasmid construction	LONP1-insert3-F	gacttgTCCAAGGTGCTGTTCATCTGC
Plasmid construction	LONP1-insert3-R	cctcgagagatctcggtcgacTCACCGTTCCACGGCCAG
Plasmid	TIGAR-site1-F	CGACGCGTaccgcacaggctaaactaattccga

construction		
Plasmid construction	TIGAR-site1-R	CCAAGCTTCCTCGTGATCCGCCCGCCTCGACCT
Plasmid construction	TIGAR-site2-F	CGACGCGTgttaaaaacggcaagactttactca
Plasmid construction	TIGAR-site2-R	CCAAGCTTTTTATCAAGAGAGACCTCACTGTTG
Plasmid construction	TIGAR-site3-F	CGACGCGTagatcgagaccatcctggctaa
Plasmid construction	TIGAR-site3-R	CCAAGCTTTTGAGATGGAGTCTGGCTCTGT
Plasmid construction	TIGAR-S3mut-F	CGACGCGTagatcgagaccatcctggctaacagtctctactaaaaataca
Plasmid construction	TIGAR-S3mut-R	CCAAGCTTTTGAGATGGAGTCTGGCTCTGTCACCCAGGCTGGAGTGCAGT
RT-PCR for ChIP	CHIP-S1RT-F	ggtggagtaggtaatgggaa
RT-PCR for ChIP	CHIP-S1RT-R	CTCGTGATCCGCCCGCCTCG
RT-PCR for ChIP	CHIP-S2RT-F	agagaaattattcatctgag
RT-PCR for ChIP	CHIP-S2RT-R	TGGCTATAAATCTCCACTGG
RT-PCR for ChIP	CHIP-S3RT-F	actttgggaggtcgaggcgg
RT-PCR for ChIP	CHIP-S3RT-R	TAGCTGGGACTACAGGCGCC
RT-PCR for ChIP	CHIP-DR5-F	AGGTTAGTTCCGGTCCCTTC
RT-PCR for ChIP	CHIP-DR5-R	CAACTGCAAATTCCACCACA
Genotyping mouse	F	ACACTGAATTACATCCGTTTCAGGGT
Genotyping mouse	R	CAATGTACCGTCTATGTGCAAGCC

Genotyping mouse	Del	CTTCACTACTCTTGACCCTGCGTCC

Table S5. Antibody Information

Proteintech Group	anti-DDIT3, anti-CHAC1, anti-STC2, anti-ASNS, anti-PCK2, anti-PINK1, anti-NDUFS1, anti-ETFDH, anti-COQ9, anti-ETFA, anti-NDUFB5, anti-SDHB, anti-SDHC, anti-FXN, anti-CYTB, anti-UQCRC1, anti-UQCRC2, anti-UQCRB, anti-UQCRQ, anti-COX1, anti-COX4, anti-COX5B, anti-COX6B1, anti-COX7A2L, anti-ATP5A1, anti-ATP5B, anti-ATP5C1, anti-ATP5D, anti-ATP5O, anti-LONP1, anti-CLpP, anti-YME1L1, anti-FATP2, anti-ASCL1a, anti-ATGL, anti-CD36, anti-HK1
Cell Signaling Technology	anti-HK2, anti-PFKL, anti-GPI, anti-ALDOA, anti-ALDOB, anti-PGK1, anti-PKM2, anti-PGAM1, anti-ENO1, anti-LDHA, anti-TPI, anti-CS, anti-ACO2, anti-IDH2, anti-OGDH, anti-S-COA, anti-SDHA, anti-FH, anti-MDH1, anti-DLD, anti-DLST, anti-IDH1, anti-DDIT3(CHIP), anti-ATF4, anti-PARP, anti-GLUT1, anti-mouse IgG(H+L), anti-rabbit IgG(H+L)
Santa Cruz	anti-p53, anti-TOMM20
Sigma-Aldrich	anti-FLAG, anti-LC3
Invitrogen	Donkey anti-Rabbit IgG (H+L) highly crossed-adsorbed secondary antibody Alexa Fluor 488, Donkey anti-Mouse gG (H+L) highly crossed-adsorbed secondary antibody Alexa Fluor 568
CMC-TAG	anti-GAPDH, anti-Actin

ABclonal	anti-OSGIN1, anti-GDF15,anti-GCN2
Abcam	anti-CLpX, anti-TIGAR
Affinity	Anti-phospho-GCN2 (Thr899)
